# Autonomous Nanomedicine: Closing the Biological Validation Gap

**DOI:** 10.1002/exp2.70228

**Published:** 2026-11-04

**Authors:** Yan Jun Lin, Izabella Komperda

**Affiliations:** ^1^ Department of Biological Sciences Cornell University Ithaca New York USA; ^2^ Department of Mechanical Engineering Rutgers University New Brunswick New Jersey USA

**Keywords:** biological validation, drug delivery, drug discovery, nanomedicine, organ‐on‐chip, protein corona

## Abstract

Although nanomedicines offer advanced formulation control and physicochemical characterization, clinical translation has been limited. Delivery meta‐analyses and late‐stage attrition indicate biological validation is often a bottleneck. It must account for context‐dependent processes such as protein corona formation, immune recognition and cell/tissue barrier effects, which standard preclinical testing cannot always assess adequately. To address this, we propose a Design–Make–Test–Biology–Learn (DMTBL) workflow that separates biological validation from physicochemical testing and feeds human‐relevant biological results back into formulation design. We assess the capabilities required for end‐to‐end autonomy using an author‐assigned technology‐readiness evaluation with explicit criteria adapted for biomedical translation. Many of the required technologies are approaching practical deployment, including high‐throughput robotic formulation, automated physicochemical assays, predictive models for biodistribution and toxicity, and patient‐derived organoids with growing human relevance. But no closed‐loop DMTBL system has yet been demonstrated: integration sits at technology readiness level 2–3 against components at levels 3–8. The Learn step also requires a different computational stack from the Analyze step it replaces, handling imaging and multi‐parametric phenotypes rather than scalar physicochemical metrics. Three practical barriers remain: mismatched timescales between formulation and biological testing, heterogeneous data combining quantitative measurements with imaging and biological variability across platforms and donors. We set out near‐term engineering and standardization priorities within a staged roadmap that separates plausible short‐term milestones from longer‐horizon goals.

## Graphical abstract

Delivery of nanoparticles to tumors has not improved in over fifteen years, and biological testing remains slow and separate from formulation design. This review proposes adding a Biology step to the design cycle, using organ chips and patient‐derived organoids, and assesses the readiness of each required technology.

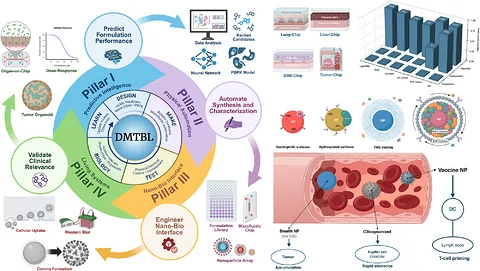

## 1 The Nanomedicine Translation Crisis

Nanomedicine has produced a vast amount of literature and numerous formulation platforms. However, few nanomedical formulations translate into approved therapeutics. Attrition of candidate therapeutics rises sharply as they move from controlled preclinical settings into complex and variable human biology. Inadequate biological validation is a major and under‐addressed bottleneck in nanomedicine translation. The limiting capability is not the generation or characterization of nanomaterials but the establishment of biological efficacy early enough to inform the design of new versions of those materials.

Nanomedicine has attracted sustained large‐scale research investment over more than 2 decades [1, 2]. Nonetheless, only a few dozen nanomedical formulations have achieved regulatory approval, and oncology nanomedicines in particular show clinical success rates among the lowest in biomedical research, well below those of conventional small‐molecule drugs.

A meta‐analysis of 117 publications quantified the magnitude of this issue: median delivery of therapeutic agents to tumors was only 0.7% of administered dose [1]. An updated meta‐analysis that included 534 datasets from 297 studies published between 2005 and 2021 confirmed this finding, reporting a median of 0.67%ID with no statistically significant improvement over time (0.74% for 2005–2018 vs. 0.52% for 2018–2021; P=0.086) [3]. As Figure 1A illustrates, clinical attrition steepens dramatically at Phase II. That pattern indicates biological complexity outpacing physicochemical optimization at the point where candidates enter patients. Accumulation at this level is generally regarded as insufficient for cytotoxic payloads, although the accumulation actually required for efficacy depends on cargo potency, release kinetics, exposure duration and tumor mass, so no single threshold applies across nanomedicines (Figure 1B). Accumulation varies across tumor types, with median delivery spanning roughly fivefold between the least and most permissive types in pooled preclinical data (Figure 1C). These variations reflect substantial heterogeneity in the enhanced permeability and retention (EPR) phenomenon that physicochemical optimization alone cannot overcome [4, 5]. The three medians quoted here—0.7%ID [1], 0.67%ID [3] and 0.76%ID [5]—come from overlapping but non‐identical dataset compilations and agree to within rounding. Price et al. questioned the validity of using percentage injected dose (%ID) as a measure of therapeutic agent delivery to tumors, arguing that the ${\mathrm{AUC}}_{\text{tumor}}$/${\mathrm{AUC}}_{\text{plasma}}$ ratio yields a median of approximately 0.4 (40% of plasma exposure). Absolute delivery remained low, but the widely cited 0.7% figure may understate tumor exposure relative to the amount circulating in plasma [6]. Administering about one trillion nanoparticles to mice overwhelmed Kupffer cells and resulted in up to 12%ID tumor delivery [7]. The 0.7% figure applies specifically to solid tumor delivery in preclinical models. mRNA‐LNP vaccines target injection sites, draining lymph nodes, and liver through a fundamentally different pharmacokinetic mechanism, and their clinical success does not alter the solid tumor delivery statistics discussed previously.

**FIGURE 1:**
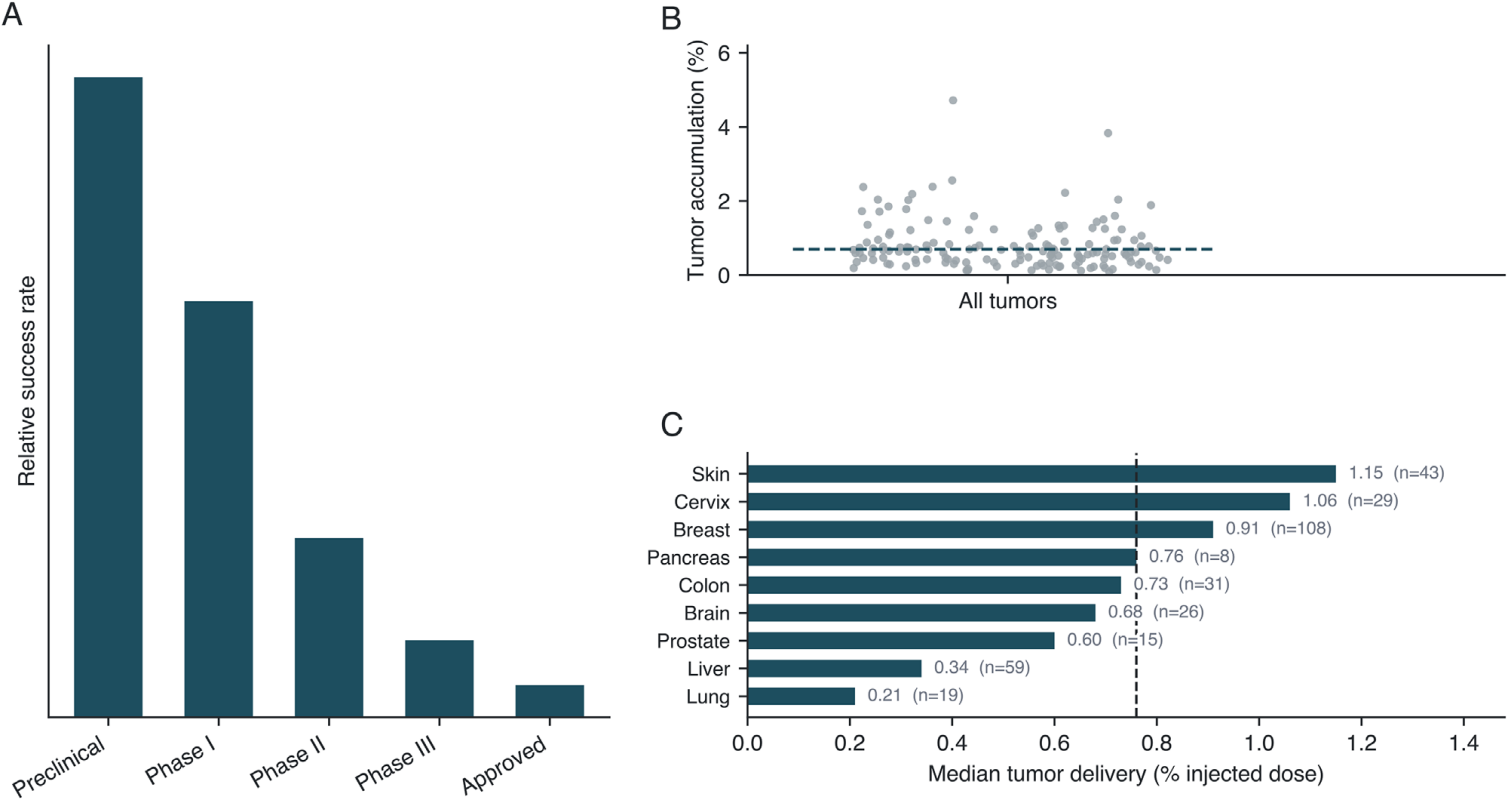
Nanomedicine translation failures. (A) Schematic of clinical attrition, showing the steep decline at Phase II. Bar heights are relative and are not drawn from a defined cohort. (B) Distribution of tumor delivery across preclinical models, with a median of 0.7%ID. An updated meta‐analysis (534 datasets, 2005–2021) confirmed a median of 0.67%ID with no significant improvement over time (P=0.086) [3]. (C) Median tumor delivery efficiency by cancer type, plotted from Cheng et al. [5] Table 1 (median ${\mathrm{DE}}_{\text{Tlast}}$, %ID; n = datasets per type). The spread across nine cancer types is approximately fivefold (0.21% –1.15%ID), against an all‐datasets median of 0.76%ID (dashed line); Wilhelm et al. [1] report concordant values. The Ouyang dose‐threshold discovery (1 trillion NPs achieving 12%ID [7]) demonstrates that this delivery barrier is an engineering challenge.

BIND‐014, a PSMA‐targeted docetaxel nanoparticle, illustrates a recurring pattern in which nanoparticles optimized for physicochemical endpoints such as tumor localization and receptor binding do not go on to deliver correspondingly large gains at biological endpoints such as intracellular release and functional delivery. BIND‐014 showed both tumor localization and PSMA binding in animal studies, and its Phase I trial reported clinical activity with acceptable tolerability [8]. Among the mechanisms that have been proposed to explain why localization of this kind does not translate into proportionate efficacy are insufficient intracellular drug release, variable receptor expression within tumors, and competitive inhibition by endogenous ligands. Similarly, CRLX101 (a camptothecin‐cyclodextrin conjugate) demonstrated excellent preclinical data but yielded disappointing Phase II results in ovarian cancer, indicating that accumulating therapeutic agents in tumors does not guarantee their efficacy as therapeutics [9].

Evidence from the approved nanomedicines indicates that Doxil (PEGylated liposomal doxorubicin) reduces cardiotoxicity compared to free doxorubicin but achieves comparable overall survival in most clinical settings [10]. Abraxane (albumin‐bound paclitaxel) improves solubility and eliminates Cremophor‐associated hypersensitivity but provides modest efficacy improvements over conventional paclitaxel [11]. Onpattro (patisiran), the first FDA‐approved siRNA therapeutic, demonstrated transformational efficacy in hereditary transthyretin amyloidosis [12] but required 2 decades of LNP optimization before achieving clinical success. Approved nanomedicines have most consistently improved pharmacokinetics, formulation or tolerability, although some, including patisiran, have produced substantial therapeutic benefit.

The mRNA‐LNP vaccine platform is among the most successful clinical demonstrations of nanomedicine to date, yet emerged from 4 decades of empirical optimization [13]. The ionizable lipid SM‐102 in Moderna's formulation and ALC‐0315 in Pfizer/BioNTech's formulation were discovered through systematic screening of hundreds of candidates. Karikó and Weissman's foundational work on nucleoside modifications required years of iterative experimentation [14]. This trial‐and‐error approach, while ultimately successful, required time and resource investments that most nanomedicine programs cannot sustain. The COVID‐19 pandemic demonstrated both the potential of nanomedicine platforms and the limitations of empirical discovery. Despite unprecedented resources and regulatory flexibility, vaccine development still required 11 months from sequence release to emergency authorization.

### 1.1 The Automation–Biology Asymmetry

Automation and self‐driving laboratories have improved formulation discovery by accelerating synthesis and characterization cycles via microfluidics and closed‐loop optimization [15, 16, 17, 18]. Platforms such as Ada [19], mobile robotic chemists [20] and the Chemputer [21] can execute reproducible experimental sequences at throughput well beyond manual workflows.

While synthesis and physicochemical characterization take hours, meaningful biological validation (organoid viability, organ‐chip pharmacokinetics) takes weeks. The gap between them is filled with multi‐fidelity Bayesian optimization with asynchronous batching [22]. Low‐fidelity surrogates (computational predictions in minutes, 2D culture cytotoxicity in 24–48 h) guide ongoing synthesis batches while high‐fidelity results (organoid or organ‐chip responses at 7–14 days) are pending. When high‐fidelity data arrives, models are recalibrated. McDonald et al. have demonstrated this approach in drug discovery by running docking (cost 0.01), single‐point biochemical assays (cost 0.2) and dose‐response curves (cost 1.0) simultaneously across all fidelities [23]. Distributed asynchronous SDL workflows further leverage idle time for parallel physicochemical characterization, computational surrogate retraining and exploration of adjacent chemical space [18]. Hardware‐level coupling between microfluidic synthesis platforms and organ‐chips enables real‐time formulation‐to‐biology integration. Effluents from a microfluidic nanoparticle synthesis chip can be routed directly into an organ‐chip's vascular channel through standard Luer‐lock or tubing connections, eliminating manual transfer steps. Tobias et al. reviewed self‐driving laboratories and their policy implications, noting that closed‐loop platforms demonstrated to date couple automated synthesis with biochemical rather than human‐relevant biological assays [24].

Therefore, nanoparticles can be optimized for physicochemical criteria (for example, size, dispersity and colloidal stability) yet still fail in vivo due to rapid corona formation [25, 26], complement activation and immune clearance [27, 28], limited functional uptake despite surface engineering [29] or toxicity caused by nano–bio interactions [30, 31]. Consequently, physicochemical characterization alone does not ensure success.

Species differences further complicate translation. For identical formulations, mouse and human protein coronas can differ substantially, with limited overlap reported in proteomics studies (Figure 2A) [32, 33]. Such discordance can shift opsonization and clearance pathways, interacting with other cross‐species gaps including EPR magnitude and complement regulation (Figure 2B,C) [4, 34]. These considerations motivate integrating human‐relevant platforms (organ‐on‐chip systems, patient‐derived organoids and human plasma profiling) earlier in discovery cycles, as summarized in Figure 2D.

**FIGURE 2:**
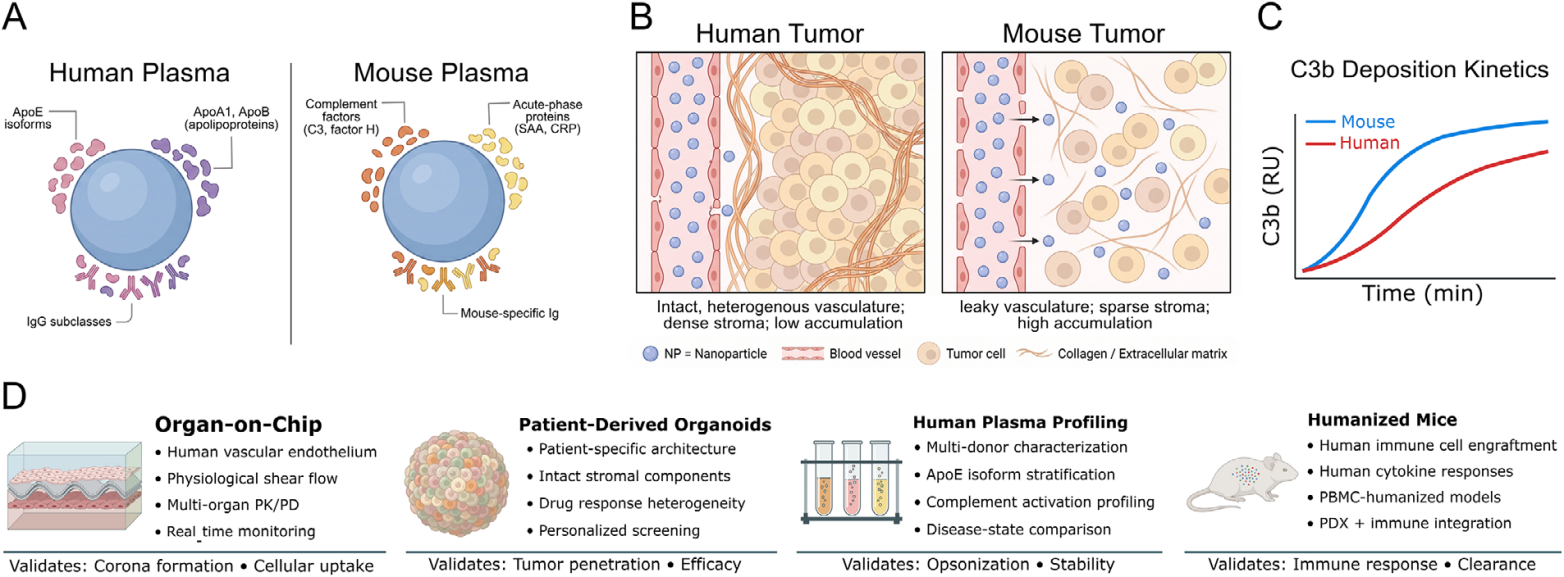
Species‐specific biology motivates human‐relevant validation. (A) Protein corona composition differs substantially between mouse and human plasma. (B) Tumor vasculature and stromal architecture differ between human tumors and mouse xenografts, a recognized contributor to cross‐species discordance in EPR‐dependent accumulation. (C) Complement activation kinetics and regulation vary by species. (D) Human‐relevant platforms for early DMTBL integration.

### 1.2 The Promise and Limitations of Predictive Intelligence

A number of machine learning models have reported strong performance on nanomedicine prediction tasks. Using LightGBM on 2664 nanoparticle records, a 10‐fold cross‐validated accuracy of 0.976 was obtained for the prediction of nanoparticle toxicity to environmental test organisms [35]. Deep neural networks trained on the 376‐dataset Nano‐Tumor Database gave an adjusted $R^{2}$ of 0.92 for maximum delivery efficiency on the training set [36]. For protein corona functional class prediction, values of $R^{2}$ above 0.80 have been reported [37]. Nano‐QSAR models have been applied to cellular uptake [38] and to mixture toxicity, where an $R^{2}$ of 0.911 was obtained [39]. Further approaches continue to be developed. Some of these use the protein corona as a meta‐descriptor [40], while others model dynamic time‐dose responses [41] or species‐specific traits [42].

However, training accuracy exceeds validation accuracy in every study compared here, though not all of those comparisons use matched metrics or independent datasets, so a pooled estimate of the size of that gap is not defensible. Prospective validation, which involves prediction of outcomes for subsequently synthesized formulations, remains rare. Dataset sizes are modest (typically <3000 formulations), limiting generalization to novel chemical space. Biological complexity spans four hierarchical scales, each of which gives rise to behaviors that cannot be predicted from measurements at lower scales alone.

*Molecular scale* (seconds–minutes): Formation of hard corona ($K_{d}<$1 nM, hours residence) and soft corona ($K_{d}>$100 nM, minutes residence) is established through competitive Vroman effects, with temporal evolution continuing for days in vivo [25, 43, 44, 45]. *Cellular scale* (hours): Uptake pathways are dependent on corona‐presented epitopes, with CRISPR screens implicating regulators such as SLC18B1 transporters, so far reported only in preprint, and NHLRC2‐RhoA‐Rac1 axes [29, 46]. *Tissue scale* (days): EPR heterogeneity exhibits substantial inter‐tumor variation, while organ‐specific barriers require distinct optimization strategies [1, 4]. *Organismal scale* (weeks): System‐level complexities are introduced by multi‐organ pharmacokinetic interactions, route‐dependent parameter sensitivity and patient‐specific genomic/proteomic variability [47].

There are also limits to what computational modeling of corona dynamics can achieve at present. Single‐protein adsorption can be captured by all‐atom molecular dynamics at nanosecond‐to‐microsecond timescales. Multi‐protein nanoparticle systems, however, remain too large to be simulated over the physiologically relevant range of minutes to hours [48]. Coarse‐grained models extend the accessible regime somewhat, to competitive binding among three protein types in a meso‐scale treatment [49]. Hybrid experiment‐simulation approaches have also been reported. In one such approach, Langmuir kinetics calibrated against experimental affinities were combined with simulation, and corona kinetics were predicted over $10^{-3}$ to $10^{3}$ s [50]. Full prediction of the Vroman effect in realistic blood plasma, where nanoparticles encounter hundreds of plasma proteins, remains beyond current computational reach [51]. For the purposes of Design–Make–Test–Biology–Learn (DMTBL) integration, ML surrogates trained on experimental corona data appear to be the more practical option. First‐principles simulation is still useful for explaining the mechanisms of particular interactions. Current automation performs well at synthesis and physicochemical characterization. The biological complexity described above, however, is not captured.

## 2 The DMTBL Framework

Traditional Design‐Make‐Test‐Analyze (DMTA) cycles, successful in materials science and small‐molecule drug discovery, prove insufficient for nanomedicine because they conflate physicochemical testing with biological validation [15, 16, 17, 52]. Here we propose a modified framework that addresses this limitation.

### 2.1 From DMTA to DMTBL: Addressing Biological Complexity

The distinction between materials science and nanomedicine lies in what “Test” measures. Materials science tests intrinsic properties (conductivity, band gap) that are context‐independent. Nanomedicine requires testing extrinsic properties (corona composition, cellular uptake) that vary with biological context. In traditional DMTA cycles, “Test” encompasses physicochemical characterization and “Analyze” denotes standard statistical evaluation; biological assessment is most often handled as a downstream evaluation step rather than as an explicit participant in the optimization loop. In the DMTBL framework we propose here, “Test” retains its physicochemical meaning, but “Biology” is elevated to a distinct, integrated node representing human‐relevant biological validation (corona formation, cellular uptake, organ‐chip efficacy, and immune activation) whose outputs feed directly into the “Learn” phase. This makes biological validation an explicitly scheduled node rather than an activity folded into “Test”, which is how it is most commonly handled in practice.

In a DMTA loop, the “Analyze” step processes scalar physicochemical metrics (hydrodynamic diameter, polydispersity index, zeta potential, encapsulation efficiency) using conventional regression or response surface methods. In DMTBL, the “Learn” step must ingest high‐dimensional biological phenotypes: morphological fingerprints from cell painting assays (hundreds of features per cell), time‐resolved organ‐chip pharmacokinetic profiles, corona proteomics mass spectra, and multiplexed organoid viability readouts. These data modalities require convolutional neural networks for image‐based phenotypes, graph neural networks for corona composition‐to‐function mapping, and multimodal fusion architectures to integrate heterogeneous biological signals into a unified objective function for the next Design iteration. The computational stack required for Learn is qualitatively different from that required for Analyze, and this difference is why DMTBL requires different engineering considerations.

Identical nanoparticle formulations acquire different biological identities in PBS, fetal bovine serum, mouse plasma, and human blood because the adsorbed protein corona, complement engagement, and overall composition depend strongly on the surrounding milieu and species [32, 33, 53]. As a result, a particle tuned to perform well in mouse tumor models can perform poorly in humans if the human corona redirects it to hepatic macrophages rather than the intended target tissue. The DMTBL framework therefore distinguishes physicochemical Testing (size, charge, encapsulation efficiency) from Biology validation (corona formation, cellular uptake, organ‐level biodistribution, immune activation), acknowledging that favorable physicochemical metrics are necessary but not sufficient for therapeutic success [54, 55].

The DMTBL framework is material‐agnostic in its logical structure, but implementation details necessarily vary across nanoparticle classes. For lipid nanoparticles and liposomes, microfluidic synthesis is well established, corona dynamics strongly influence biodistribution, and organ‐chip integration is most mature. Polymeric nanoparticles (PLGA, micelles) are amenable to microfluidic synthesis but involve longer degradation timescales requiring extended biological validation windows. Inorganic nanoparticles (gold, iron oxide, silica) present different toxicity endpoints centered on oxidative stress and persistent biopersistence, and their PBPK models require distinct compartmental parameters. Biological nanoparticles (exosomes, cell membrane‐coated particles) possess inherent biological identity, making corona formation less relevant but introducing different “Test” and “Biology” endpoints related to cargo loading and immunogenicity. The framework's value is structural: biological data must close the loop, regardless of which assays produce it. Corona profiling applies to any nanoparticle class exposed to biological fluids, while microfluidic synthesis serves as a shared manufacturing backbone across lipid and polymeric systems; these methodologies converge at the nano‐bio interface regardless of the starting material.

Figure 3 presents the complete DMTBL architecture organized across four integrated pillars. At the center, the Design–Make–Test–Biology–Learn cycle drives iterative optimization, with each pillar contributing distinct capabilities. Pillar I (predictive intelligence) employs AI/ML prediction, nano‐QSAR modeling, and PBPK simulations to guide formulation design, represented by neural network architectures and ranked candidate outputs. Pillar II (physical automation) executes robotic synthesis through microfluidic platforms, generating formulation libraries and nanoparticle arrays with automated characterization. Pillar III (nano‐bio interface) addresses the critical physicochemical‐to‐biological transition through corona formation analysis and complement profiling. Pillar IV (living systems) provides human‐relevant biological validation using organ‐on‐chip platforms, tumor organoids, and dose‐response characterization to establish clinical relevance. The outer arrows emphasize the closed‐loop nature: biological outcomes continuously update predictive models, enabling iterative refinement toward formulations with improved translational potential.

**FIGURE 3:**
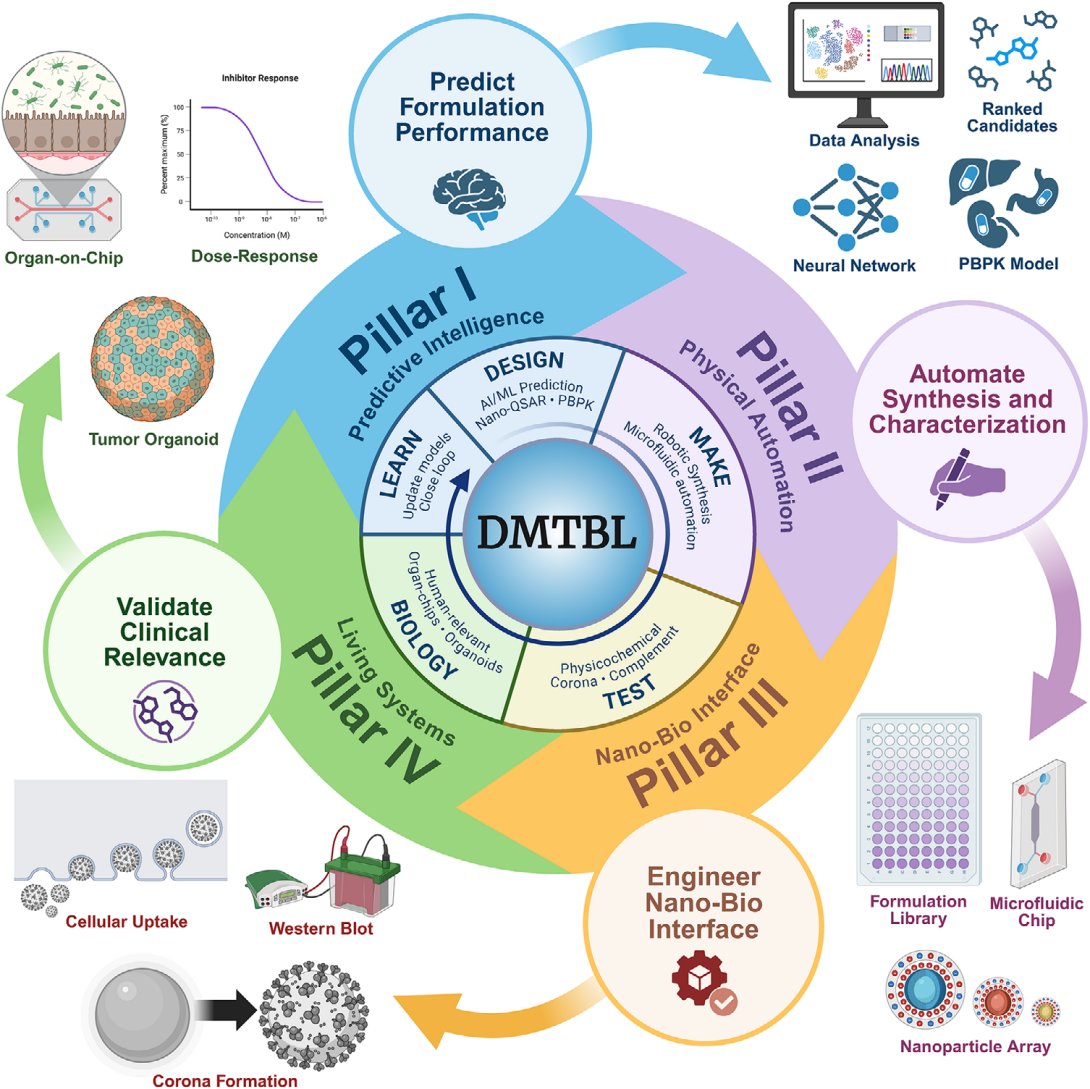
The DMTBL framework integrates four pillars for autonomous nanomedicine discovery. The central Design–Make–Test–Biology‐Learn cycle coordinates Pillar I (predictive intelligence: AI/ML, nano‐QSAR, PBPK modeling for formulation prediction), Pillar II (physical automation: microfluidic synthesis, robotic characterization, formulation libraries), Pillar III (nano‐bio interface: protein corona dynamics, complement profiling, physicochemical‐biological transition), and Pillar IV (living systems: organ‐on‐chip, tumor organoids, dose‐response validation for clinical relevance). Outer arrows illustrate closed‐loop integration: biological outcomes continuously update predictive models, enabling iterative optimization from design through validation.

### 2.2 Hierarchical Biological Validation Across Four Scales

Biological validation must traverse four hierarchical scales, each with its own timescales, mechanisms, and emergent properties. Figure 4A shows molecular‐level corona dynamics; Panel B contrasts the traditional DMTA cycle with the DMTBL framework proposed here.

**FIGURE 4:**
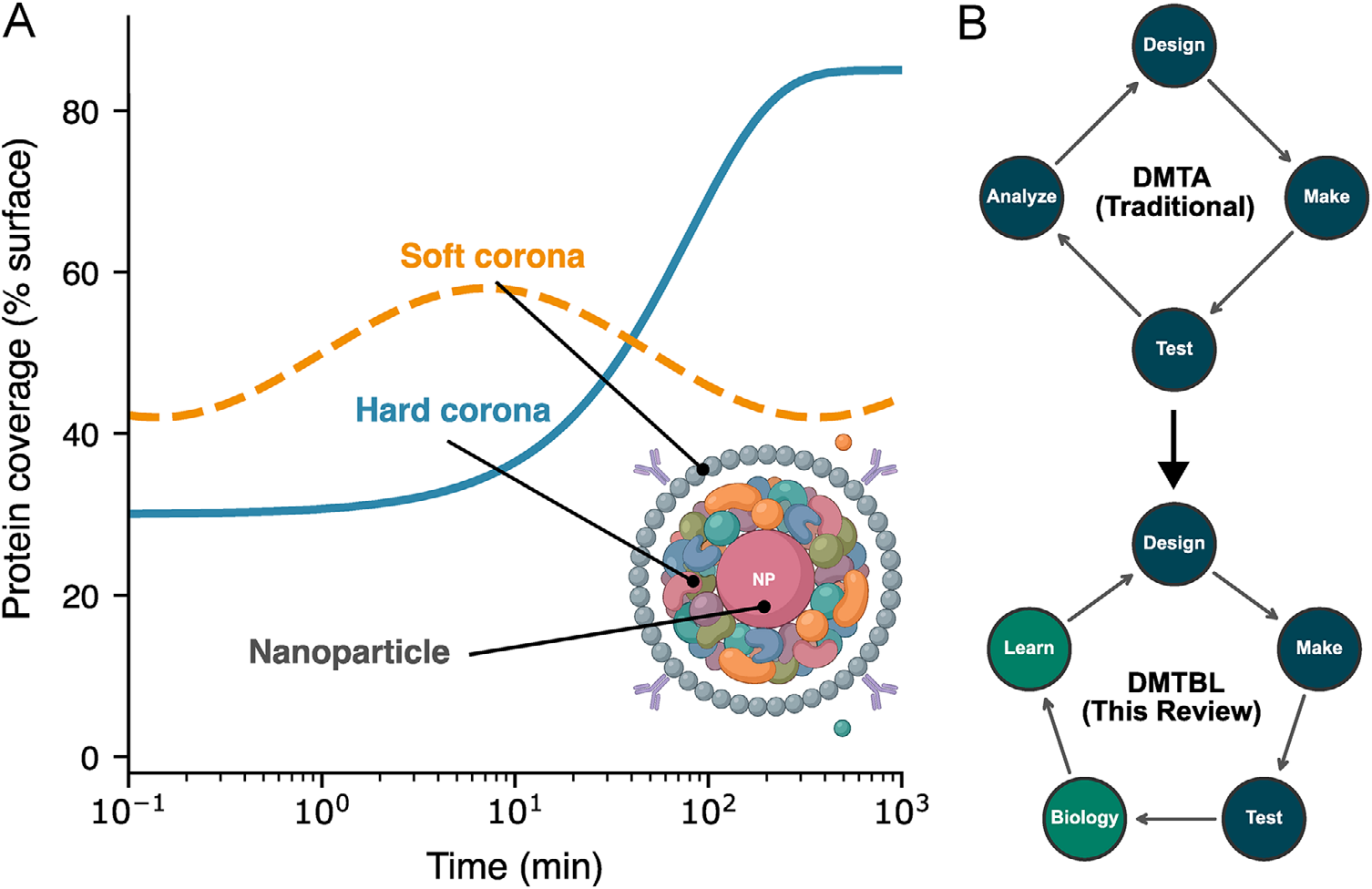
Biological complexity across scales and the DMTBL response. (A) Molecular scale: corona formation defines nanoparticle identity within minutes, with hard corona (blue) and soft corona (orange) following distinct kinetics. (B) The DMTBL framework addresses this complexity by separating physicochemical Testing from biological validation (Biology) and closing the optimization loop through a Learn phase that feeds human‐relevant biological data back to Design, a structural advance over traditional DMTA cycles, which evaluate biology downstream and outside the loop.

At the *molecular scale* (0.5–60 min), protein corona formation defines nanoparticle identity, and it does so quickly. As shown in Figure 4A, hard corona proteins (albumin, fibrinogen, IgG; $K_{d}<$1 nM) bind with residence times of several hours or longer. Soft corona proteins (vitronectin, complement factors; $K_{d}>$100 nM), by contrast, undergo dynamic Vroman‐type exchange on the timescale of minutes [25, 43]. Surface chemistry is a major determinant of which complement pathway is engaged. Surface chemistry strongly affects complement engagement, but the direction is formulation‐dependent. In polypropylene sulfide nanoparticles, polyhydroxylated surfaces covalently capture the exposed C3b thioester and promote complement activation [27]. Factor H recruitment and the C3b→iC3b conversion depend on surface architecture and cannot be assigned universally to hydroxylated surfaces. Corona formation can also be stereospecific: chiral nanoparticles have been reported to recruit distinct protein profiles depending on whether the L‐ or D‐enantiomer is presented [44].

At the *cellular scale* (1–24 h), uptake is governed less by the bare surface chemistry of the particle than by the epitopes that the corona presents. A genome‐wide CRISPR screen, reported in preprint and not yet peer‐reviewed, identified SLC18B1, a previously orphan vesicular polyamine transporter, as required for polymeric micelle uptake through a pathway apparently unrelated to classical endocytosis [29]. NHLRC2 has also been implicated, and it regulates phagocytosis through RhoA‐Rac1‐actin cytoskeletal remodeling [46].

At the *tissue scale* (1–7 days), EPR heterogeneity varies considerably across tumor types, and pooled preclinical data place the median delivery for most common tumor types below 1.2% of injected dose [4, 5]. Organ‐specific barriers impose additional constraints: the tight junctions of the blood–brain barrier restrict transport, tumor stromal matrices physically impede penetration beyond the perivascular region, and the mononuclear phagocyte system removes a large fraction of the injected dose from the circulation within hours [1]. Microphysiological systems have revealed behaviors that are simply not visible in static culture. In one such study, a lung‐on‐chip model showed that cyclic mechanical stretch from breathing increased the translocation of silica nanoparticles across the alveolar‐capillary interface, an effect that was attributed to mechanosensitive ion channels [56].

At the *organismal scale* (1–4 weeks), system‐level complexity emerges from multi‐organ pharmacokinetic interactions, route‐dependent parameter sensitivity, and inter‐individual variability. PBPK models incorporating intravenous, oral, intratracheal, and inhalation routes indicated that the parameters to which predictions are most sensitive differ by administration route [47, 57]. Temporal dynamics compound these difficulties further. The in vivo protein corona evolves continuously from minutes to days after injection, and the opsonin composition on circulating nanomaterials is remodeled over time rather than remaining fixed [45]. Patient‐specific factors, including genetic polymorphisms in complement factors, apolipoprotein isoforms (ApoE2/E3/E4), and disease‐induced proteomic alterations, give rise to personalized corona compositions that standard preclinical models are not able to capture [58].

## 3 Technology Readiness Assessment

A practical evaluation of autonomous nanomedicine requires separating what has been demonstrated from what remains a longer‐horizon aspiration. We adapted the technology readiness level (TRL) framework to a biomedical context to do this. Laboratory, relevant environment, and operational environment demonstration settings were mapped onto preclinical, translational, and clinical stages, respectively. Most component technologies across the DMTBL pillars sit at mid‐to‐high TRLs. Fully integrated, end‐to‐end workflows do not; they remain, for the most part, at an early stage.

### 3.1 Component‐Level Readiness

#### 3.1.1 Predictive Intelligence (TRL 3–5)

Pillar I was assigned a lower TRL range than its reported performance metrics alone might suggest. This is intentional. Retrospective model fitting is not the same thing as prospective predictive utility, and we wanted the TRL range to reflect that distinction rather than the raw numbers reported in the literature (see Table 1 for our adapted TRL criteria). Most nano‐QSAR models operate at TRL 3: retrospective cross‐validation on existing datasets. PBPK models generally do a bit better. When animal‐derived parameters are validated against held‐out data, they reach TRL 4, component validation in a laboratory environment. TRL 5 is more demanding. Validation in a relevant environment requires prospective testing on novel formulations synthesized after the model was already built, and none of the six studies summarized in Table 2 reports validation of that kind. Corona prediction can reach $R^{2}$ above 0.80 for functional class prediction [37], though performance on the harder task of predicting individual protein abundances is markedly lower [59]. Many approaches, moreover, represent only a single static snapshot rather than tracking how the corona composition changes over time. Generative design models are able to propose synthesizable candidates, but the experimentally synthesized and tested subset remains a small fraction of the computationally generated library [60].

**TABLE 1:** Technology readiness level definitions adapted for biomedical and nanomedicine contexts. Adapted from HHS PHEMCE and VA Biomedical TRL frameworks. TRL assignments for DMTBL pillars reflect prospective validation requirements.

TRL	Generic definition	Nanomedicine adaptation	DMTBL pillar status
1	Basic principles observed	Target/mechanism identified; initial computational studies	—
2	Technology concept formulated	Initial nano‐QSAR descriptors defined; proof‐of‐concept models	—
3	Proof of concept	Retrospective model validation on existing datasets (cross‐validation)	Pillar I: most nano‐QSAR models
4	Lab validation	Component validated in controlled lab; external validation on held‐out data	Pillar I: PBPK with animal data; Pillar III
5	Validation in relevant environment	Prospective prediction of novel formulations; validated against subsequent synthesis	Pillar I: absent (0/6 studies); Pillar IV
6	Demonstration in relevant environment	Integrated workflow demonstrated; predictions validated in human‐relevant platforms	Pillar II: microfluidic synthesis
7	System prototype in operational environment	Pre‐IND regulatory submission using model; organ‐chip data supporting efficacy	Pillar IV: limited
8	System complete and qualified	Clinical trial enrollment using DMTBL‐guided formulations	Pillar II: commercial platforms
9	Operational deployment	Approved nanomedicine developed via autonomous pipeline	None yet achieved

**TABLE 2:** Reported machine learning performance across nanomedicine prediction tasks. Values are quoted as reported in each source. Only two of six report any form of independent validation, and none reports prospective validation on formulations synthesized after the model was built.

Task	Dataset	Reported performance	Independent validation	Prospective	Ref.
Ecotoxicity classification (LightGBM)^a^	2664 records	Accuracy 0.976 (10‐fold CV)	Not reported	Not reported	[35]
Tumor delivery (DNN)	376 datasets	$R^{2}$=0.92 (${\mathrm{DE}}_{\max}$, training)	Not reported	Not reported	[36]
Tumor delivery (AI‐PBPK)	376 datasets	$R^{2}$=0.83 (${\mathrm{DE}}_{24}$); 0.82 (${\mathrm{DE}}_{\max}$); 0.56 (${\mathrm{DE}}_{168}$)	Not reported	Not reported	[61]
Corona functional composition (RF)	652 data points	$R^{2}>$0.80 for most protein classes	Not reported	Not reported	[37]
Corona relative protein abundance (ERT)	178 proteins / 652 points	AUROC 0.969; accuracy 0.919 (classification)	$R^{2}\approx$ 0.50 on 178 held‐out proteins (regression)^b^	Not reported	[59]
Mixture toxicity (NN)	7 metallic ENPs, 22 binary combinations	$R_{\text{test}}^{2}=0.911$ (internal)	$R_{\text{test}}^{2}=\;0.908$ (internal + external)	Not reported	[39]

a Environmental test organisms in nutrient solution.

b Classification and regression performance diverge sharply on the same dataset; the regression figure is the held‐out result.

#### 3.1.2 Physical Automation (TRL 6–8)

Microfluidic synthesis platforms are already commercially deployed. Formulation libraries can be produced at scale [62, 63]. Self‐driving laboratories have pushed this further, automating synthesis and physicochemical characterization at high throughput [19, 20]. Automated characterization methods, DLS and zeta potential measurements among them, can be run in plate formats with electronic‐record compliance [64]. Biological assays lag behind. Their integration into these automated loops is considerably less mature.

#### 3.1.3 Nano–Bio Interface Engineering (TRL 4–5)

Strategies such as dysopsonin pre‐coating have been shown to improve stealth under controlled conditions [65]. Batch reproducibility remains a challenge. So does variability across different patients' plasma backgrounds. Active targeting runs into a related problem: only a small fraction of conjugated targeting ligands adopts an orientation competent for receptor binding, as has been reported for transferrin‐grafted conjugates [66].

#### 3.1.4 Living Systems Integration (TRL 5–7)

Organ‐chip and organoid platforms have shown improved human relevance for specific endpoints in published validation studies, hepatotoxicity prediction and patient‐matched drug response among them [67, 68, 69]. Policy and regulatory interest in non‐animal methods is increasing. Performance and standardization, however, still vary considerably across platforms and endpoints. Inter‐platform variability can exceed 15% CV for certain readouts. Cross‐manufacturer standardization remains incomplete. One report puts inter‐laboratory variation in organoid viability testing in China at 40% [70], and first‐pass metabolism rate variations of up to 30% have been observed across multi‐organ chip configurations [71].

### 3.2 The Integration Gap

Published demonstrations, on the whole, stop short of a complete closed‐loop cycle in which models propose candidates conditioned on biological performance, robotic platforms synthesize and characterize candidates, human‐relevant systems return biological validation data, and those outcomes update models for iterative optimization.

Partial closed‐loop workflows exist. Even so, they often rely on simplified biological validation. Bateni et al., for instance, reported a self‐driving fluidic lab that autonomously discovered record‐performance doped quantum dots within a single day of closed‐loop experimentation [72]. This underscores how quickly machine‐learning‐guided synthesis can navigate a complex parameter space. The Novartis MicroCycle platform, which upgraded an automated high‐throughput synthesis and characterization system into a self‐driving laboratory, likewise relies on biochemical rather than human‐relevant biological endpoints [24]. By contrast, high‐throughput organoid or chip screening often functions as a downstream evaluation step rather than as feedback for generative design.

Three constraints continue to limit integration. Each, though, has a near‐term mitigation strategy.

#### 3.2.1 Barrier 1: Temporal Mismatch

Synthesis completes in hours, while biological validations demand days to weeks. Multi‐fidelity Bayesian optimization with asynchronous batching offers a direct answer to this [22, 23]. Low‐fidelity surrogates (computational predictions in minutes; 2D culture at 24–48 h) supply immediate guidance for ongoing synthesis. Mid‐fidelity organ‐chip data (1–2 weeks) and high‐fidelity organoid data (2–4 weeks), by comparison, arrive asynchronously and recalibrate the models at each tier. The SDL never idles: wait time goes toward parallel physicochemical characterization, surrogate model retraining, and exploration of adjacent chemical space using fast proxies [18]. The temporal asymmetry here is pronounced: a traditional DMTA cycle completes synthesis (4 h), physicochemical characterization (2 h), and statistical analysis (1 h) within a single day, but the subsequent animal study requires 2–4 weeks. In a DMTBL cycle built around multi‐fidelity optimization, by contrast, the same synthesis and characterization occur on day 1, computational surrogates return predictions on day 1, 2D cytotoxicity data arrives on days 2–3, organ‐chip readouts on days 7–14, and organoid responses on days 14–28, with each tier recalibrating the model asynchronously. By the time the high‐fidelity biological data arrives, the SDL has already worked through 3–5 additional formulation batches guided by the lower‐fidelity feedback. Figure 5A depicts the fidelity cascade, and Figure 5B juxtaposes the two workflows.

**FIGURE 5:**
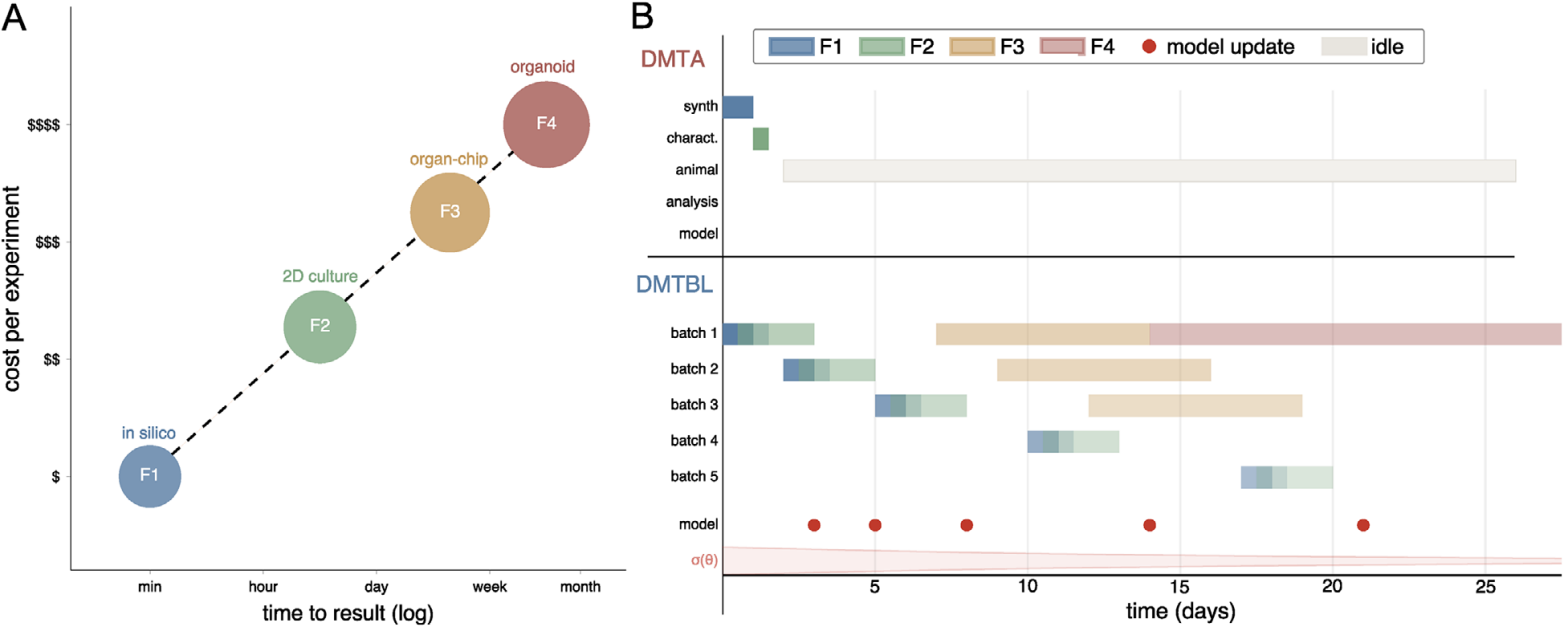
Multi‐fidelity asynchronous optimization closes the DMTBL loop despite the synthesis‐biology timescale gap. (A) Four validation fidelities (F1 in silico through F4 patient‐derived organoid) span approximately four orders of magnitude in per‐experiment cost and time‐to‐result; predictive relevance to human biology increases monotonically with fidelity. (B) Schematic comparison over an identical 30‐day budget, illustrating scheduling logic rather than the output of a simulation. Serial DMTA (top) completes a single formulation, with the platform idle while a terminal high‐fidelity readout is awaited. DMTBL (bottom) stages five batches and runs F1–F4 assays in parallel, so that model recalibration (red dots) recurs within the same window; the shaded band indicates the intended direction of change in parameter uncertainty σ(θ) and is not a computed posterior. Conceptual framework after Folch et al. [22]; turnaround times after McDonald et al. [23] and organ‐chip/organoid literature.

#### 3.2.2 Barrier 2: Data Heterogeneity

Biological assays yield images and multi‐parametric phenotypes that are not immediately represented as structured model inputs. Solutions include: (a) Cell Painting combined with convolutional neural networks to convert high‐dimensional morphological fingerprints into structured feature vectors; (b) FAIR‐compliant ontologies (NPO, CHEBI, ENPO) that standardize diverse readouts into machine‐readable formats [73]; and (c) knowledge graphs linking synthesis parameters, characterization data, and biological outcomes through graph neural network architectures. Figure 6C maps out this data pipeline.

**FIGURE 6:**
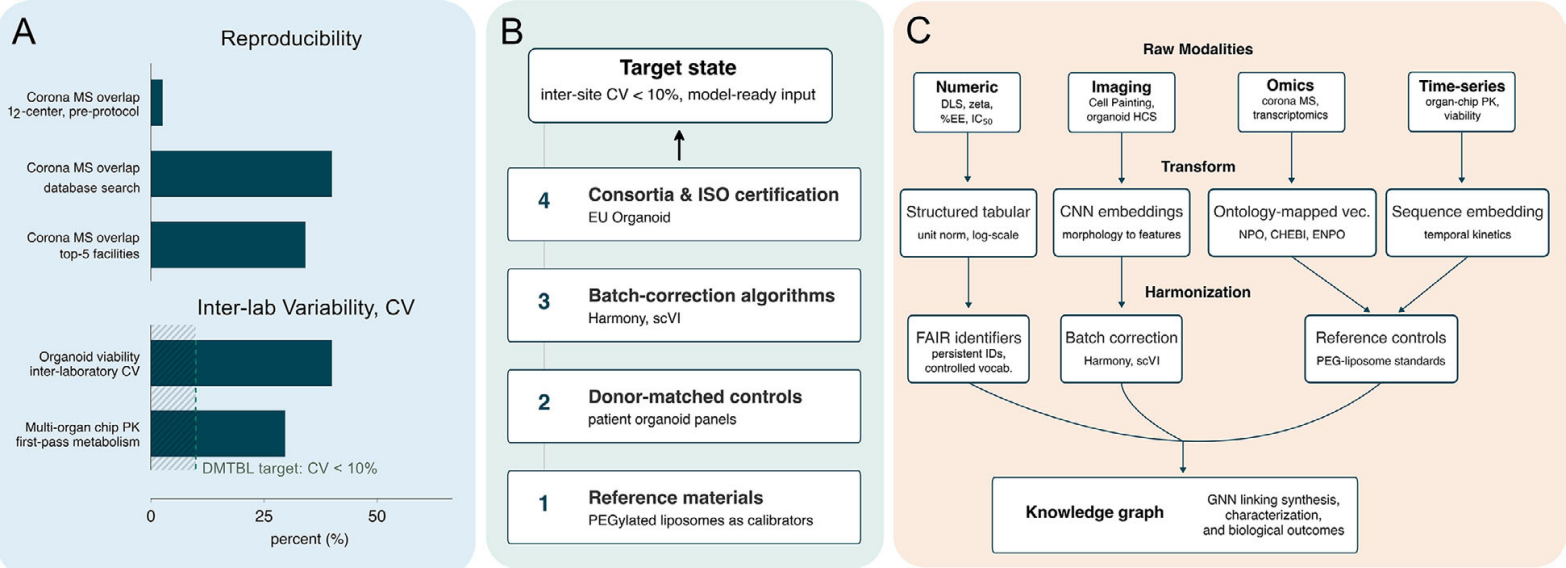
Biological data heterogeneity is the rate‐limiting constraint for DMTBL. (A) Variability in biological ground truth across core DMTBL readouts. A 12‐center proteomics study found only 1.8% consistent protein identification; applying a uniform database search raised this to 35.3% among the top five facilities and 16.2% among the top eleven [74]. Additionally standardizing sample preparation and instrumentation raised overlap from 11% to 40% across facilities using comparable instruments [75]. Reported inter‐laboratory organoid viability variation reaches 40% in a Chinese multi‐site survey [70], and multi‐organ‐chip first‐pass metabolism varies by up to 30% [71]. The green dashed threshold marks the inter‐site CV <10% target for scalable DMTBL deployment. (B) Four mitigation layers required before biological data can reliably close the optimization loop, ordered from most local (per‐lab reference standards) to most systemic (international consortia). (C) Concrete data‐pipeline architecture addressing Barrier 2: four raw biological modalities are passed through modality‐specific transforms (tabular normalization, CNN morphological embeddings, ontology‐mapped vectors using NPO/CHEBI/ENPO, and sequence embeddings), harmonized via FAIR identifiers, batch‐correction algorithms (Harmony, scVI), and reference‐control anchoring (PEG‐liposome standards), then integrated into a knowledge graph queried by a graph neural network linking synthesis parameters, characterization data, and biological outcomes.

#### 3.2.3 Barrier 3: Biological Variability

Inter‐platform variation can mask formulation‐dependent effects unless assay designs and standards are improved (Figure 6B). Mitigation strategies include: (a) standardized reference formulations (e.g., well‐characterized PEGylated liposomes) as inter‐platform calibrators; (b) donor‐matched controls using patient organoid panels with matched plasma for corona formation; (c) batch correction algorithms borrowed from single‐cell RNA‐seq analysis (Harmony, scVI) to harmonize readouts across platforms; and (d) coordinated standardization initiatives, such as the EU “Organoid” initiative, which has improved data comparability by applying ISO‐style acceptance criteria including >80% cell viability and <5% genomic instability [70].

No published study has yet demonstrated that SDL‐optimized nanomedicine formulations achieve superior clinical outcomes compared to conventionally developed comparators. The nearest approximations are Bateni et al.'s autonomous discovery of record‐performance quantum dots [72] and the Novartis MicroCycle platform [24]. Both used physicochemical or biochemical endpoints rather than human‐relevant biology or clinical efficacy. Until at least one DMTBL‐optimized formulation demonstrably outperforms a conventional comparator in a human‐relevant validation platform, the framework's translational value remains a testable hypothesis rather than an established fact.

### 3.3 Biological Data Standardization as a Rate‐Limiting Constraint

The DMTBL loop cannot converge if the ground‐truth biological data feeding the Learn step is noisy and non‐reproducible (Figure 6A). The magnitude of this challenge is evident in protein corona characterization work. A multi‐center study in which identical corona samples were analyzed by 12 proteomics facilities found only 1.8% overlap in consistently identified proteins; applying a uniform database search raised consistency to 35.3% of proteins shared among the top five facilities and 16.2% among the top eleven [74]. A further study that additionally standardized sample preparation and instrumentation increased overlap from 11% to 40% across facilities using comparable instruments [75]. That is considerably better, though still far from convergent. Mahmoudi and colleagues have proposed comprehensive standardized protocols across corona formation, isolation, and mass spectrometry characterization steps to further improve reproducibility [76].

Organ‐chip platforms confront similar difficulties: inter‐laboratory variation in organoid viability testing has been reported at 40% in China [70], and first‐pass metabolism rate variations of up to 30% have been observed across multi‐organ chip configurations [71]. These variability levels outstrip the signal‐to‐noise ratio needed for meaningful model training. Inter‐site coefficient of variation below 15% is the ceiling currently accepted for commercial organ‐chip standardization; we propose below 10% for key biological endpoints as a working target for scalable DMTBL deployment. Doing so will require consensus protocols, reference materials, and inter‐laboratory proficiency testing programs.

## 4 Quantitative Synthesis

### 4.1 Machine Learning Performance

Comparison across the studies selected here reveals several patterns across prediction tasks (Table 2). The most consistent finding is not the size of any performance gap but its *unmeasurability*: four of the six studies report no independent validation at all, so no training‐versus‐validation comparison can be made. Where independent validation is reported, the picture is mixed. Zhang et al. lose almost nothing when external data are added ($R_{\text{test}}^{2}$ 0.911 → 0.908), whereas Fu et al. show a sharp divergence on a single dataset: classification of corona membership reaches AUROC 0.969, while regression of relative protein abundance on 178 held‐out proteins falls to $R^{2}\approx$0.50. That contrast is instructive, since headline metrics are typically drawn from the easier task. Applicability domain violations, where models are asked to predict for chemical spaces outside their training distribution, are not routinely reported in nanomedicine ML studies. The highest reported accuracy (LightGBM, 0.976 on 2664 records) should be interpreted cautiously: it is a 10‐fold cross‐validation figure rather than a held‐out result, and the endpoint is ecotoxicological rather than mammalian. Prospective validation, the gold standard in which models predict outcomes for subsequently synthesized formulations, is not reported in any of the six studies summarized here. Dataset sizes cluster around 500–3000 examples, substantially smaller than the 10,000+ records typical for successful drug discovery ML applications.

### 4.2 Biological Platform Validity

Living systems platforms report improved predictive validity relative to conventional preclinical models, whose concordance with human toxicity is incomplete: 71% for rodent and non‐rodent species combined, 63% for non‐rodent and 43% for rodent species alone [77]. However, only three platforms report a sensitivity/specificity pair against a defined benchmark (Table 3). Liver cancer organoids, zebrafish, and *Caenorhabditis elegans* screening are omitted from that comparison because none reports such a pair; they are used for drug‐sensitivity profiling and rapid triage rather than for predictive validity [78, 79, 80]. The small number of validation studies makes it difficult to evaluate the predictive validity of living systems platforms. The platforms illustrated in Figure 7 achieve this improved validity through recapitulation of human‐specific physiology: organ‐chips incorporate mechanical forces and tissue–tissue interfaces absent in static cultures, while patient organoids preserve tumor architecture and genetic heterogeneity.

**TABLE 3:** Reported predictive validity of biological platforms. Sensitivity and specificity are tabulated only where the primary source reports both. Prospective validation against clinical outcomes is absent for every platform listed.

Platform	Sens.	Spec.	Benchmark	Endpoint and caveats
Liver‐Chip (Emulate)	87%	100%	18 drugs [68]	Hepatotoxicity; 15 toxic drugs; sensitivity 95% CI 61.5–96.0%
3D hepatic spheroids	47%	100%	22 drugs [68]	Hepatotoxicity; previously published spheroid data, reanalyzed with protein‐binding correction
GI cancer organoids	100%	93%	110 biopsies, 71 patients [69]	Treatment response; 88% PPV, 100% NPV
Traditional 2D	Not systematically benchmarked for nanomedicine endpoints			
Animal models	71% concordance with human toxicity (63% non‐rodent, 43% rodent) [77]; ILSI survey of 150 compounds across 12 companies, reporting target‐organ concordance rather than a sensitivity/specificity pair			

**FIGURE 7:**
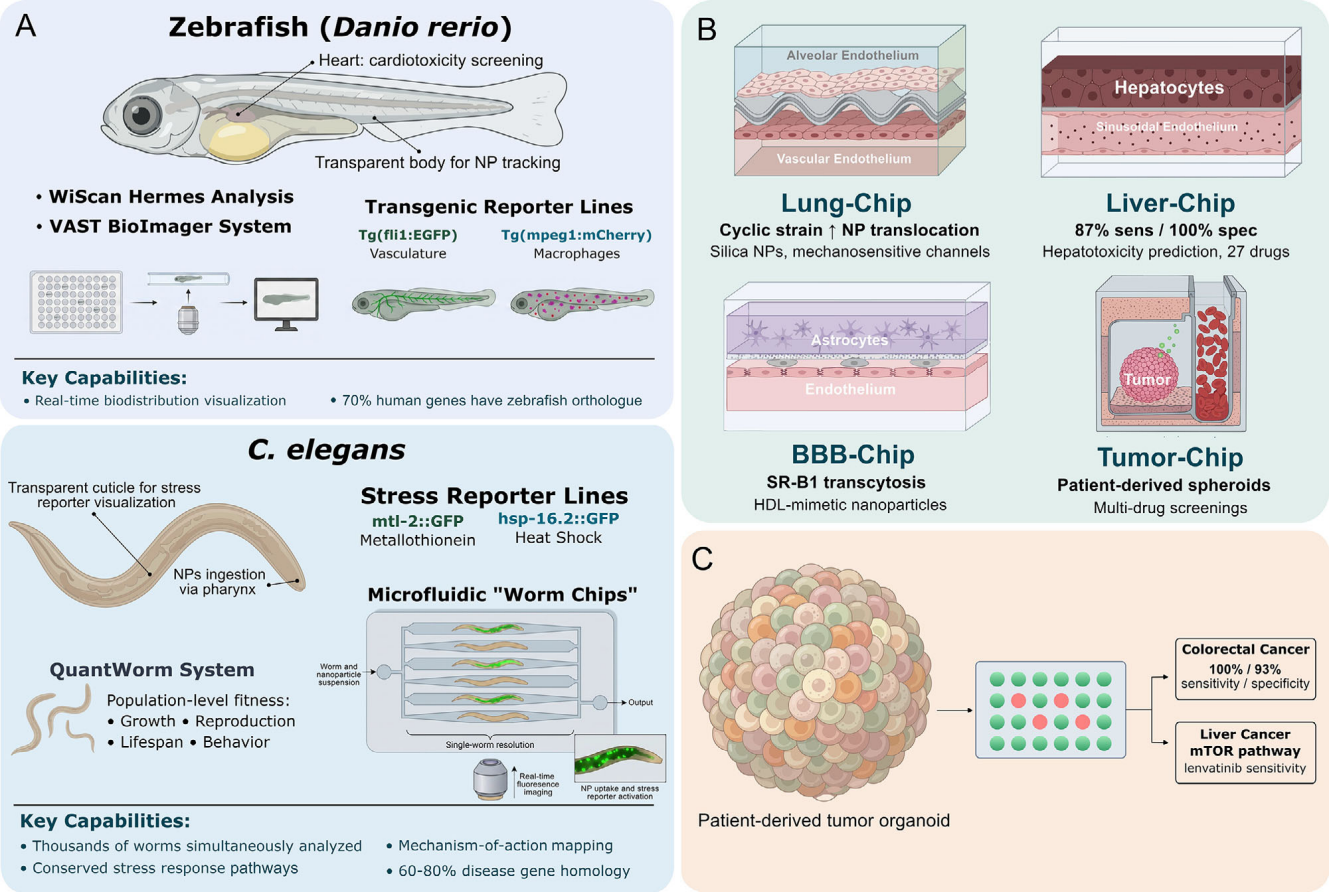
Tiered biological validation platforms. (A) Tier 1: Model organisms (zebrafish, *C. elegans*) enable rapid whole‐organism toxicity screening, biodistribution imaging, and MPS clearance kinetics. These organisms are *not* human‐relevant for protein corona or immune specificity but provide whole‐organism dynamics that organ‐chips cannot capture, serving as a rapid triage step before committing scarce organ‐chip and organoid resources. (B) Tier 2: Organ‐chips recreate tissue interfaces and mechanical forces; regulatory interest in non‐animal methods is increasing. (C) Tier 3: Patient organoids preserve tumor architecture and forecast response to targeted agents and chemotherapy, with reported 100% sensitivity and 93% specificity in colorectal cancer.

However, the relationship between surrogate endpoints employed in these platforms and long‐term clinical outcomes is partial and hinges on context. Liver‐Chip hepatotoxicity detection attains 87% sensitivity and 100% specificity [68], but that performance does not extend past hepatotoxicity. Comparable validation for efficacy endpoints and other organ systems remains comparatively scarce. Kidney‐Chip, Heart‐Chip, and Intestine‐Chip platforms are available but have not yielded published nanomedicine‐specific validation datasets on the scale of the Liver‐Chip's 27‐drug benchmark. That gap introduces a single‐organ bias into the evidence base and constrains generalizability claims. Patient‐derived organoids have been reported to model treatment response in metastatic gastrointestinal cancers, though on the basis of a single cohort [69]. Cytotoxicity ${\mathrm{IC}}_{50}$ values, the most commonly applied surrogate endpoint, poorly predict chronic effects such as fibrosis, immunogenicity, and organ accumulation. Corona functional protein classes can be predicted from nanoparticle properties with $R^{2}$ above 0.80 [37], but no comparable accuracy has been demonstrated for the step from corona composition to biodistribution, and the predictive chain from in vitro or organ‐chip measurements to long‐term clinical outcomes necessitates large‐scale prospective validation studies that have not yet been conducted. This is the single largest evidentiary gap holding back DMTBL. No study has shown that corona composition, organ‐chip pharmacokinetics, or organoid drug response quantitatively predicts long‐term safety and efficacy across diverse nanomedicine classes. Without such evidence, surrogate endpoints used in the Biology node remain unvalidated proxies for clinical outcomes.

## 5 Four Pillars of Autonomous Nanomedicine

### 5.1 Pillar I: Predictive Intelligence

Predictive intelligence provides the mathematical foundation linking formulation design to biological outcomes. Practically, this pillar exists to rank candidates before they are ever made, using data‐driven and mechanistic models; it quantifies uncertainty, translates findings across species and platforms, and in doing so points experiments toward whichever formulations are most informative. Figure 8 maps the key computational approaches: physiologically‐based pharmacokinetic modeling, machine learning workflows, corona‐aware prediction, generative design architectures.

**FIGURE 8:**
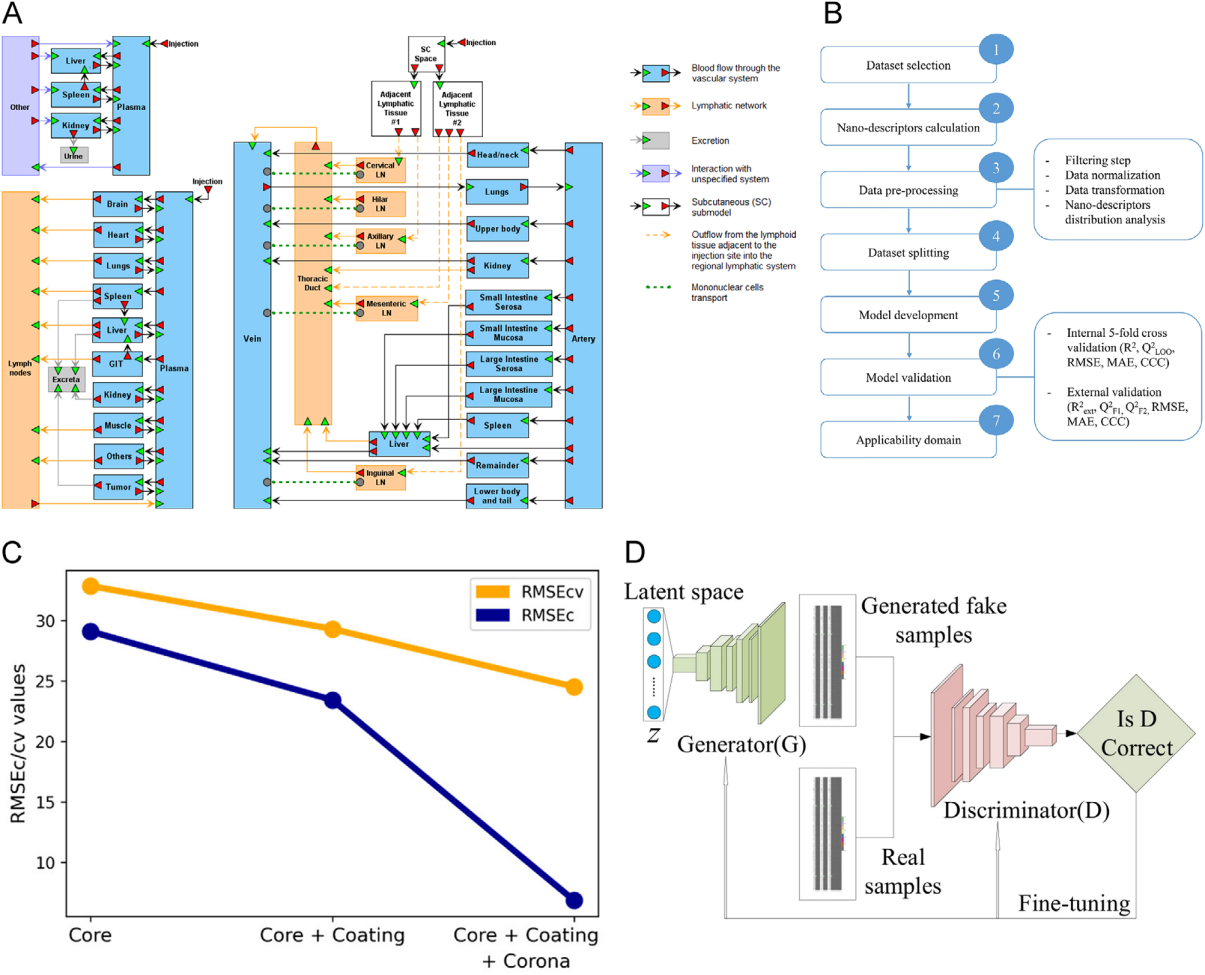
Pillar I: Predictive intelligence framework for autonomous nanomedicine. (A) Physiologically‐based pharmacokinetic (PBPK) compartmental models for nanoparticle biodistribution showing intravenous and subcutaneous administration routes with organ‐specific distribution. Adapted under the terms of the CC BY 4.0 license [81]. Copyright 2022, The Authors, published by MDPI. (B) Nano‐QSTR modeling workflow illustrating the seven‐step pipeline from dataset selection through model validation and applicability domain assessment. Adapted under the terms of the CC BY 4.0 license [82]. Copyright 2023, The Authors, published by Springer Nature. (C) Impact of protein corona on nano‐QSPR prediction accuracy demonstrating RMSE reduction when incorporating core, coating, and corona descriptors. Adapted under the terms of the CC BY 4.0 license [40]. Copyright 2023, The Authors, published by American Chemical Society. (D) Generative adversarial network (GAN) architecture for materials design showing latent space sampling, generator network, discriminator evaluation, and fine‐tuning cycle. Adapted under the terms of the CC BY 4.0 license [83]. Copyright 2020, The Authors, published by Springer Nature.

#### 5.1.1 Nano‐QSAR, Machine Learning, and Toxicity Prediction

Nano‐QSAR models have matured from pioneering applications [84] to sophisticated ensemble methods reaching a cross‐validated accuracy of 0.976 via LightGBM on 2664 nanoparticle toxicity records, albeit for ecotoxicological rather than mammalian endpoints [35]. Early work established nano‐descriptors and QSAR methodology for quantitative nanostructure‐activity relationships [85], later refined by subsequent studies through mechanistic insight tying surface reactivity to oxidative stress [86]. Puzyn et al. set out validation criteria for nano‐(Q)SAR models covering applicability domains, mechanistic interpretability and external validation [87]. Metal oxide nanomaterial toxicity prediction moved forward through neural network architectures with SMOTE resampling for class imbalance [88, 89].

Recent innovations fold protein corona composition into the descriptor set itself: the corona governs biological response [40]. As shown in Figure 8C, incorporating corona descriptors improves prediction accuracy: calibration error (RMSEc) decreases from approximately 29 (core only) to 24 (core + coating) to 7 (core + coating + corona), with cross‐validation error (RMSEcv) tracing a parallel but shallower decline from approximately 33 to 25. Corona composition is a key determinant of nanoparticle behavior in biological systems. Moncho et al. reviewed the structural characterization methods that robust nano‐QSAR descriptors depend on [90]; on the informatics side, Jeliazkova et al. put forward standardized approaches for representing nanomaterials, complete with ontology frameworks (NPO, CHEBI, ENPO) [73, 91]. Dynamic nano‐QSAR approaches tie together time‐dose‐property relationships, predicting in vivo genotoxicity and inflammation trajectories rather than static endpoints [41]. The seven‐step nano‐QSTR workflow depicted in Figure 8B, spanning dataset selection, nano‐descriptor calculation, preprocessing, data splitting, model development, validation ($R^{2}$/$Q^{2}$/RMSE/MAE/CCC metrics), and applicability domain assessment, recorded $R^{2}$= 0.95 for training and 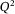
= 0.79 for external validation using human lung cell data [82]. Mixture toxicity prediction using neural networks reached $R^{2}$= 0.911 [39].

Comparative analyses suggest task‐dependent optimal architectures; ensemble methods, in general, outperform single models [92, 93]. Li et al. comprehensively reviewed nano‐QSAR modeling for metallic and metal oxide nanoparticle cytotoxicity [94]. Stacking ensemble approaches exhibited improved generalization across species‐specific datasets [42]. Recent work integrated oxidative stress, genotoxicity, and inflammatory endpoints into unified multi‐task learning frameworks [38].

#### 5.1.2 PBPK Modeling and Biodistribution Prediction

Physiologically‐based pharmacokinetic modeling has evolved from compartmental frameworks to AI‐augmented predictive systems. Figure 8A depicts the full PBPK architecture for nanoparticle biodistribution, illustrating compartmental models for both intravenous and subcutaneous administration routes with blood flow (vascular system), lymphatic network, excretion pathways, and tissue‐specific distribution including liver, spleen, kidney, brain, heart, lungs, and tumor compartments [81]. Recent reviews document advances in organ‐specific distribution modeling and MPS‐mediated clearance prediction [47], while multi‐route PBPK models now account for intravenous, oral, and inhalation exposures with validated predictions against in vivo gold nanoparticle data [57, 95]. Meta‐analysis using PBPK pegged tumor delivery as log‐normally distributed, with the 0.7% median shown in Figure 1B [5].

Deep neural network models trained on the 376‐dataset Nano‐Tumor Database reached an adjusted $R^{2}$ of 0.92 for maximum delivery efficiency on the full training set, with lower values for the other endpoints (0.76–0.77) [36]; a subsequent AI‐PBPK implementation reported $R^{2}$ = 0.83 for delivery efficiency at 24 h [61]. Multi‐view learning approaches predict multiple pharmacokinetic parameters simultaneously, pooling size distributions, charge heterogeneity, and release kinetics [61, 96]. AI‐assisted PBPK has been put to use for personalized dosing [97, 98], while multi‐route models pointed to route‐dependent parameter sensitivities, with phagocytic uptake capacity in the lung dominating predictions after intratracheal and inhalation administration [97]. Systems biology integration [81] and MCMC uncertainty quantification [99] further strengthen PBPK reliability for DMTBL deployment.

#### 5.1.3 Protein Corona Prediction, Foundation Models, and Generative Design

Protein corona prediction has moved from qualitative recognition to quantitative machine learning models. Machine learning applied to 652 curated nanoparticle‐corona data points predicted functional protein classes with $R^{2}$ above 0.80 for most classes [37]. Duan et al. developed novel descriptors that reflect surface energy landscapes [100]. Huzar et al. demonstrated DNA‐based machine learning for corona prediction [101]. Canchola et al. compiled wide‐ranging databases linking corona compositions with biological outcomes [102]. Extra‐Trees ensemble methods have since been applied to predict the relative abundance of individual plasma proteins within the corona [59]. These models identify dysopsonins (clusterin, ApoA1) and opsonins (C3, IgG) enabling rational surface engineering. Walkey et al. demonstrated that protein corona “fingerprints” predict cellular interactions more accurately than physicochemical properties [53]. Casals et al. characterized time‐evolved coronas [103]. Dell'Orco et al. modeled competitive protein binding using kinetic Monte Carlo simulations [104]. Species‐specific translation remains challenging given the corona differences shown in Figure 2A; dysopsonin pre‐coating strategies address this gap [65]. Glass et al. developed a multiphysics pharmacokinetic model for targeted nanoparticles [105].

Foundation models enable transfer learning to nanomedicine. AlphaFold‐predicted protein structures [106] have informed protein‐nanoparticle binding studies [107, 108], with molecular dynamics revealing that small gold nanoparticles induce secondary structure transitions in adsorbed proteins [48]. Separately, cryo‐EM resolved a model GroEL–platinum nanoparticle complex to 3.93 Å, demonstrating the feasibility of near‐atomic reconstruction of protein–nanoparticle interfaces [109, 110]. Chemical foundation models (MoLFormer trained on 1.1 billion SMILES strings [111]; ChemBERTa‐2 [112]) and multimodal biomedical AI [108, 113] provide few‐shot learning and cross‐domain transfer relevant to nanoparticle surface chemistry prediction.

Generative adversarial networks and variational autoencoders enable de novo design of nanoparticle formulations. Figure 8D illustrates the MatGAN architecture for materials generation, showing how a latent space vector z feeds into a generator network producing candidate structures, which are then evaluated by a discriminator network distinguishing generated “fake” samples from real training examples through iterative fine‐tuning [83]. Wang et al. computationally generated a large ionizable lipid library, screened it using gradient‐boosting models with SHAP‐guided fragment selection, and synthesized nine candidates in the pKa 6.0–7.0 window associated with efficient mRNA delivery [60]. Alakhdar et al. employed diffusion models for molecular generation [114]. Gómez‐Bombarelli et al. pioneered variational autoencoders for molecular design [115]. Generative models trained on failed formulations learn inverse relationships [83]. Fung et al. demonstrated invertible neural networks enabling bidirectional mapping [116]. Active learning workflows iteratively refine models through Bayesian optimization [117, 118, 119, 120]. Bateni et al. demonstrated closed‐loop autonomous discovery of record‐performance nanocrystals within a single day using a self‐driving fluidic lab [72].

### 5.2 Pillar II: Physical Automation

Physical automation provides the experimental backbone that realizes model‐suggested designs at useful speed and scale. This pillar transforms what would otherwise be thousands of human‐hours of pipetting and characterization into programmable, reproducible workflows that can interface with AI in near real time. Figure 9 presents the key automation technologies enabling autonomous nanomedicine discovery.

**FIGURE 9:**
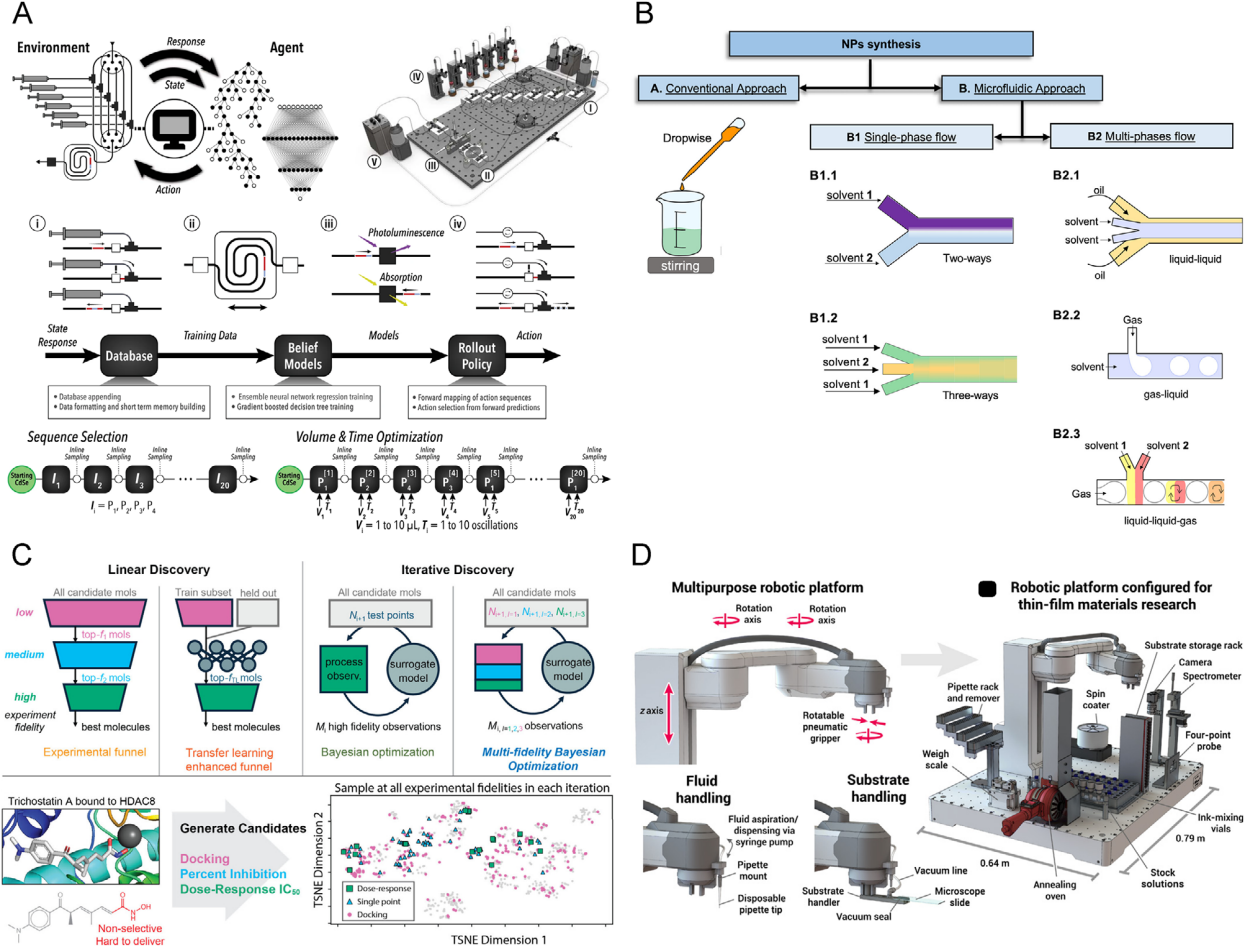
Pillar II: Physical automation for autonomous nanomedicine synthesis. (A) AlphaFlow self‐driving fluidic laboratory employing reinforcement learning for autonomous multi‐step chemistry discovery, showing database, belief models, rollout policy, and six‐module continuous flow reactor. Adapted under the terms of the CC BY 4.0 license [15]. Copyright 2023, The Authors, published by Springer Nature. (B) Microfluidic device architectures comparing conventional mixing with single‐phase and multi‐phase flow geometries for nanoparticle synthesis. Adapted under the terms of the CC BY 4.0 license [121]. Copyright 2023, The Authors, published by American Chemical Society. (C) Multi‐fidelity Bayesian optimization framework contrasting linear versus iterative discovery approaches with t‐SNE visualization of fidelity integration and docking‐to‐${\mathrm{IC}}_{50}$ cascade. Adapted under the terms of the CC BY 4.0 license [23]. Copyright 2025, The Authors, published by American Chemical Society. (D) Ada self‐driving laboratory platform for thin‐film materials discovery featuring polar robotic arm, pneumatic gripper, and automated fluid/substrate handling systems. Adapted with permission [19]. Copyright 2020, American Association for the Advancement of Science.

#### 5.2.1 Microfluidic Synthesis and Self‐Driving Laboratory Frameworks

Microfluidic platforms have advanced nanoparticle synthesis through precise control of mixing kinetics, temperature gradients, and residence time distributions. Figure 9B provides a comprehensive overview of microfluidic device architectures for nanoparticle synthesis and performance evaluation [121]. ALiS (Automated Library Synthesis) enables walk‐away processing of up to 96 unique LNP formulations in a working day, with monodispersity and encapsulation efficiency maintained across the library [62]. PreciGenome's scalable platform spans discovery through GMP manufacturing, maintaining parameters across 5 orders of magnitude [122]. TAMARA achieves sample processing in under 2 minutes with automated microfluidic mixing [123]. Historical advances established foundational principles: Zhigaltsev et al. demonstrated millisecond mixing for limit‐size LNP synthesis [124], while Leung et al. revealed that mixing rate dictates internal nanostructure [125]. LIBRIS uses eight parallel microscale mixers with lithographically encoded fluidic resistors to produce distinct LNP formulations at roughly 1000–1200 per hour, about one every four seconds, and was validated on a 96‐formulation library [63]. Gimondi et al. comprehensively reviewed microfluidic device architectures [121]. Integration with in‐line characterization enables real‐time quality assessment and closed‐loop feedback.

Self‐driving laboratories integrate robotic automation, in‐line characterization, machine learning optimization, and autonomous decision‐making [17, 18, 126]. Häse et al. articulated a vision for next‐generation experimentation [127]. Figure 9A illustrates the AlphaFlow platform, which employs reinforcement learning to guide autonomous multi‐step chemistry discovery [15]. Figure 9D shows the Ada platform for accelerated thin‐film materials discovery [19]. Ada optimized photovoltaic materials through 100+ experiments without human intervention. The mobile robotic chemist executed 688 experiments over 8 days [20]. The Chemputer employs chemical programming language (χDL) translating procedures into robotic instructions [21, 128]. Bayesian optimization drives autonomous experimental design [118]. Christensen et al. demonstrated data‐science‐driven process optimization [129]. Materials Acceleration Platforms distribute experimentation across networked facilities [130]. Frey et al. demonstrated neural scaling laws for chemical language models, suggesting that model performance improves predictably with scale [131]. Autonomy levels range from human‐in‐the‐loop (Level 1) to full autonomy (Level 5), with current nanomedicine platforms at Level 2–3 [17].

#### 5.2.2 Multi‐Fidelity Optimization, Characterization, and GMP Manufacturing

Multi‐fidelity optimization enables efficient exploration of nanomedicine design space. Figure 9C illustrates the multi‐fidelity Bayesian optimization framework for drug discovery [23]. The approach contrasts linear discovery with iterative multi‐fidelity discovery, enabling efficient allocation of experimental resources across computational docking, biochemical assays, cell‐based screens, and in vivo studies. Dynamic light scattering can be run in automated plate formats to quantify hydrodynamic diameter and zeta potential [64]. Data infrastructure challenges include FAIR compliance with persistent identifiers and standardized metadata [73]. Laboratory information management systems capture environmental conditions and reagent lot numbers. Knowledge graphs link synthesis parameters, characterization data, and biological outcomes. caNanoLab provides standardized data repositories [132].

GMP automation confronts fundamental challenges. Facility qualification is capital‐intensive and slow relative to the discovery cycles it must eventually support. Batch‐to‐batch reproducibility (CV < 5%) proves difficult for nanomedicines sensitive to sub‐degree temperature fluctuations, and scale‐up from laboratory to commercial manufacturing frequently requires reformulation. Continuous manufacturing via microfluidic numbering‐up offers alternatives, with commercial systems producing kilograms per day of mRNA‐LNPs.

### 5.3 Pillar III: Nano‐Bio Interface Engineering

In nano‐bio interface engineering, material properties are converted into the biological identities that are actually encountered by cells and tissues, since a particle that enters the blood is coated in proteins within seconds and cells respond to that coated object rather than to the bare synthetic surface. This pillar is concerned with how coronas, complement, and cell membranes interact with nanoparticles, so that circulation, uptake, and immunity can be adjusted on purpose rather than being left to chance. Advances in the understanding of protein corona composition, corona dynamics, and the functional consequences for biodistribution and targeting are shown in Figure 10.

**FIGURE 10:**
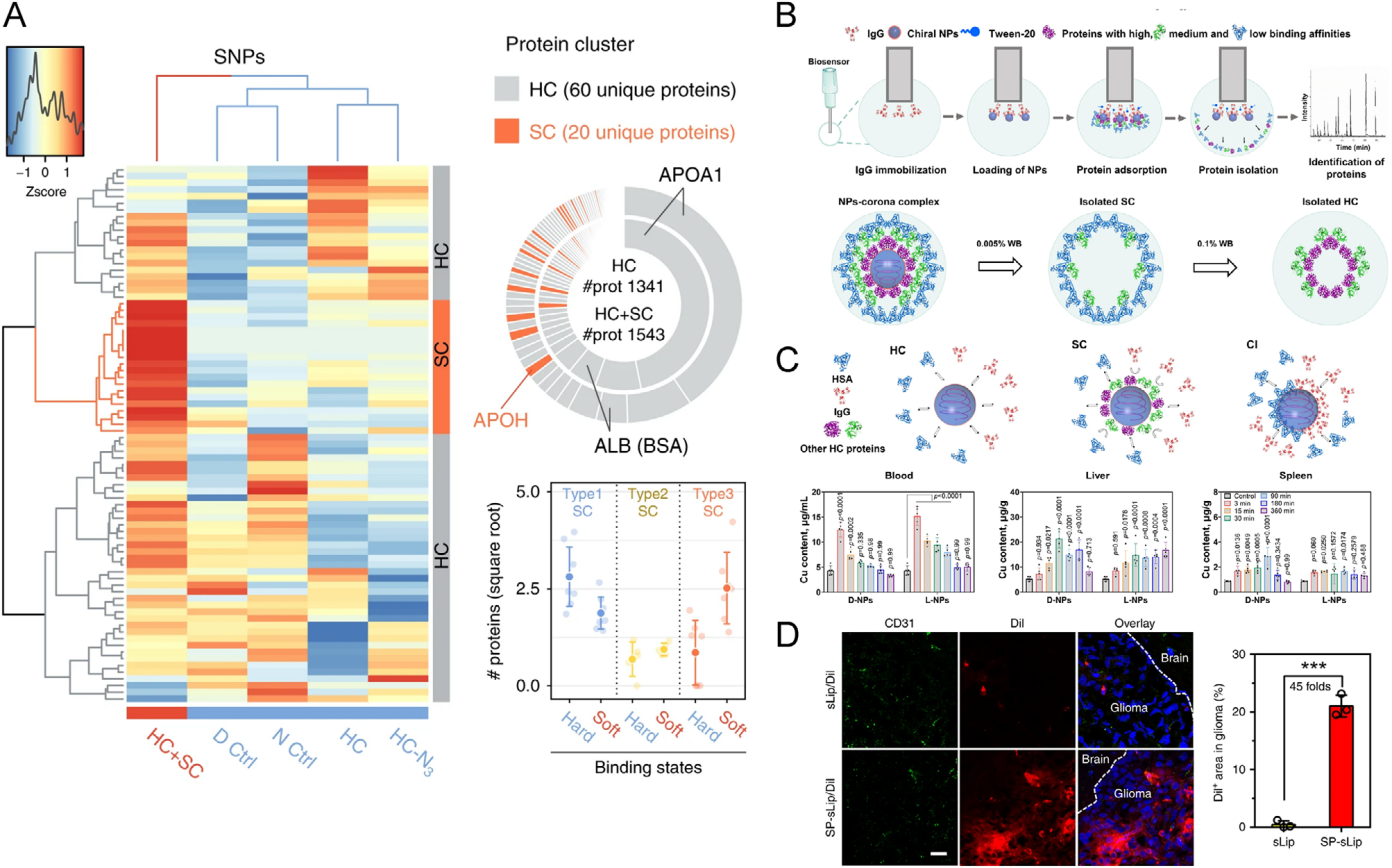
Pillar III: Nano‐bio interface engineering and protein corona dynamics. (A) Soft corona protein classification showing Z‐score heatmap with hierarchical clustering identifying Type 1/2/3 binding states and key proteins (APOA1, APOH, ALB) affecting cellular association. Adapted under the terms of the CC BY 4.0 license [43]. Copyright 2020, The Authors, published by Springer Nature. (B) Bio‐layer interferometry (BLI) “fishing” method for in situ soft corona analysis: IgG immobilization, nanoparticle loading, protein adsorption, isolation, and mass spectrometry identification distinguishing soft (0.005% WB) from hard corona (0.1% WB). Adapted under the terms of the CC BY 4.0 license [44]. Copyright 2022, The Authors, published by Springer Nature. (C) Biodistribution consequences of corona composition showing time‐course copper content in blood, liver, and spleen (3–360 min) for hard corona, soft corona, and complete interface conditions with chiral nanoparticle comparison (D‐NPs vs. L‐NPs). Adapted under the terms of the CC BY 4.0 license [44]. Copyright 2022, The Authors, published by Springer Nature. (D) Brain targeting via engineered protein corona: CD31/DiI immunofluorescence demonstrating a 45‐fold increase in glioma‐region accumulation using SP‐sLip formulations exploiting apolipoprotein‐mediated BBB transcytosis. Adapted under the terms of the CC BY 4.0 license [133]. Copyright 2019, The Authors, published by Springer Nature.

#### 5.3.1 Protein Corona Dynamics, Complement Modulation, and Uptake Mechanisms

The transformation of biological identity is brought about by the formation of the protein corona, as shown in Figure 4A [30, 55, 134]. Within 0.5 min of exposure to blood, hundreds of proteins are adsorbed by nanoparticles, and these proteins form tightly bound hard corona ($K_{d}$ <1 nM, hours‐days residence) and loosely bound soft corona ($K_{d}$ > 100 nM, minutes residence) layers [25, 26, 43]. The classification of soft corona proteins, and the effect that these proteins have on cellular association, is shown in Figure 10A, which presents Z‐score heatmaps, in which each protein is scored by how far its abundance departs from the average, with hierarchical clustering used to distinguish binding patterns across formulations [43]. Mohammad‐Beigi et al. showed that soft corona proteins function as “caretakers” that modulate cellular association even though they are only loosely attached [43]. Conformational changes upon adsorption depend on particle size: 5 nm gold nanoparticles cause more severe distortion of albumin than 50 nm particles do, and this exposes cryptic epitopes, normally buried within the folded protein, that trigger immune recognition [135, 136]. Separately, cryo‐EM resolved a model GroEL–platinum nanoparticle complex to 3.93 Å, demonstrating the feasibility of near‐atomic reconstruction of protein–nanoparticle interfaces [109]. Competitive binding through the Vroman effect, in which abundant proteins adsorb first and are displaced later by proteins that bind more strongly, is predicted by molecular dynamics simulations [48, 49]. The bio‐layer interferometry (BLI) “fishing” method that is used for in situ analysis of the soft corona, meaning analysis without first separating the particles from the biological fluid, is shown in Figure 10B [44]. IgG is immobilized on biosensor tips, nanoparticles are loaded, proteins are adsorbed from biological fluids, the corona‐coated particles are isolated, and the proteins are identified by mass spectrometry. The soft corona (isolated using 0.005% washing buffer) is distinguished from the hard corona (isolated using 0.1% washing buffer), since the concentration of washing buffer used during isolation determines which of the two layers is recovered.

Surface chemistry is linked to in vivo fate by complement activation, through the pathways that are detailed in Figure 11. The complement system, a cascade of plasma proteins that marks foreign surfaces for clearance, is a major determinant of nanoparticle biodistribution, clearance kinetics, and therapeutic efficacy. As shown in panel A of Figure 11, the classical, alternative, and lectin pathways all converge on the formation of C3 convertase, the enzyme complex that cleaves C3 and amplifies the cascade. Panel B shows how these interactions are tuned by the surface chemistry of the nanoparticle, with substantial consequences for biological fate. Nucleophilic surfaces containing amines, thiols, or carboxylic acids bind C3 covalently through the formation of a thioester bond, which favors activation of the alternative pathway and gives immunostimulatory formulations suitable for vaccines. The direction of this effect is formulation‐dependent: in polypropylene sulfide nanoparticles, polyhydroxylated surfaces covalently capture the exposed C3b thioester and promote complement activation [27], so Factor H recruitment and C3b→iC3b conversion depend on surface architecture and cannot be assigned universally to hydroxylated surfaces. PEG coatings reduce the overall adsorption of complement while enriching dysopsonins such as ApoE, which discourage rather than promote uptake by phagocytes, creating a corona favorable for extended circulation and potential brain targeting. The biological consequences of these interactions are shown in panel C.

**FIGURE 11:**
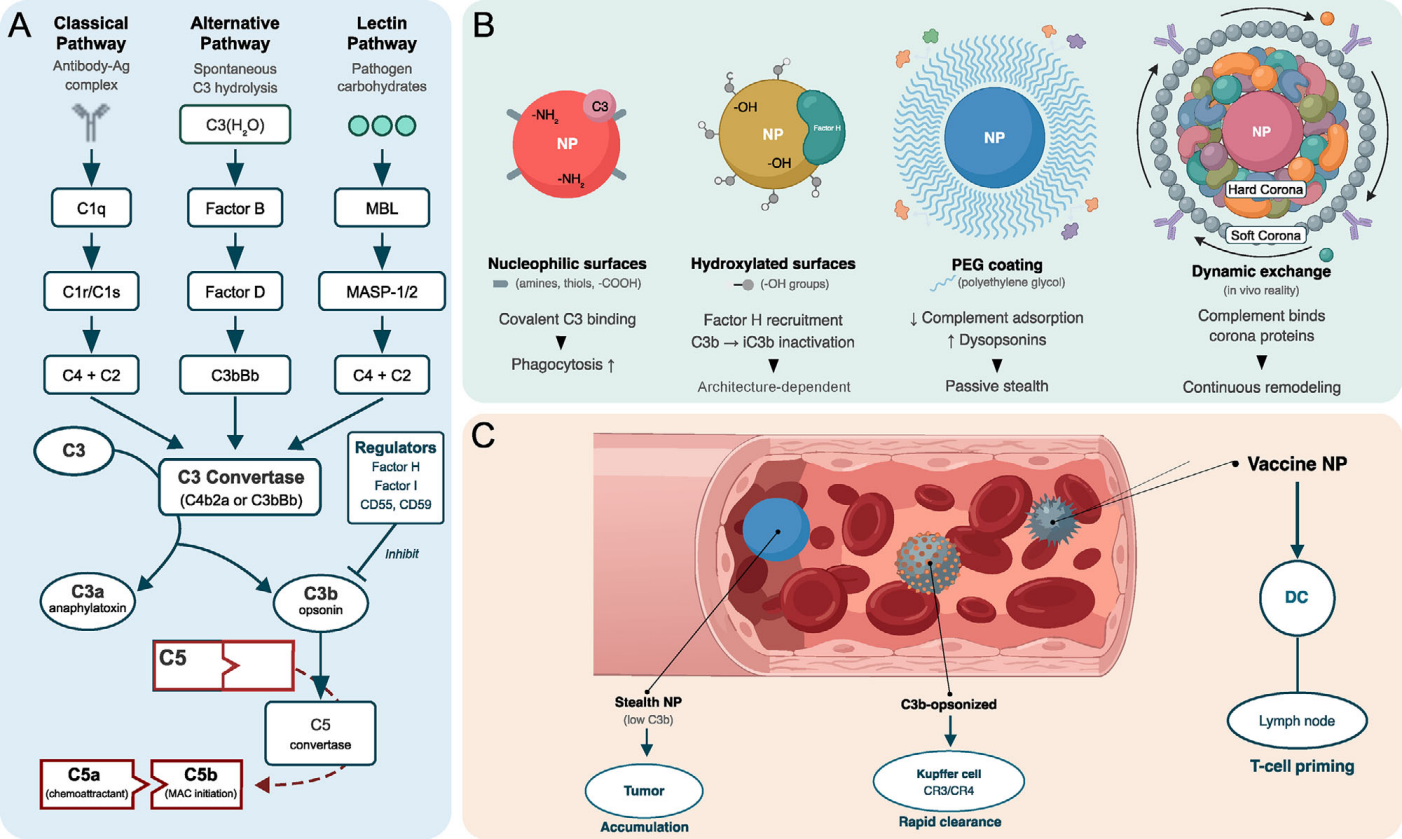
Complement activation determines in vivo fate. (A) Classical, alternative, and lectin pathways converge on C3 convertase formation, with downstream cleavage of C5 initiating MAC assembly. (B) Nanoparticle surface chemistry governs complement engagement: nucleophilic surfaces covalently bind C3, hydroxyl‐rich surfaces can recruit Factor H and drive C3b→iC3b inactivation, PEG coatings enrich dysopsonins, and dynamic corona exchange remodels the protein interface continuously in vivo. The outcome is architecture‐dependent rather than set by surface chemistry alone: polyhydroxylated polypropylene sulfide nanoparticles instead capture the C3b thioester and activate complement [27]. (C) Complement‐mediated biological fate: stealth nanoparticles with low C3b accumulate in tumors, C3b‐opsonized particles undergo rapid Kupffer cell clearance via CR3/CR4, and vaccine nanoparticles are captured by dendritic cells for lymph node T‐cell priming. Species‐specific differences in complement regulation necessitate optimizing surface chemistry for the target organism.

These design principles are supported by experimental validation. IONP‐COOH, an iron oxide nanoparticle bearing carboxyl groups, was found to activate the alternative pathway, to enhance macrophage phagocytosis, and to reduce acute myeloid leukemia burden in murine models, through improved immune recognition of the tumor cells [137]. Chen et al. showed that complement proteins bind corona proteins rather than binding nanoparticle surfaces directly, and that they undergo continuous exchange in vivo [28]. Natural antibodies, which are present in the circulation without prior exposure to the particle, trigger C3 opsonization, that is, the tagging of particles for recognition by phagocytes, through IgG‐mediated classical pathway activation [138]. Different nanoparticle types activate distinct complement cascades, which can be measured using ELISA panels [34]. La‐Beck et al. reviewed nanoparticle‐induced complement activation and its implications for cancer nanomedicine, including the protumor roles of complement within the tumor microenvironment [139]. PEG coatings decrease the adsorption of complement proteins while enriching dysopsonins such as ApoE [140]. Unexpected regulators of uptake have been identified by genome‐wide CRISPR screens, in which genes are disabled one at a time and the effect on uptake is measured. Van Nostrand et al. reported SLC18B1 as required for the internalization of polymeric micelles, in a preprint that has not been peer‐reviewed [29]. Subsequent screens identified NHLRC2, which regulates phagocytosis through RhoA‐Rac1 remodeling of the cytoskeleton, TM2D1/2/3, which give substrate‐selective uptake, and NPRL2/Ragulator/RRAGA mTOR signaling, which controls lysosomal trafficking [46]. Ritz et al. showed that this regulation is protein‐specific: internalization is decreased by ApoA4 and C3, while uptake is increased by ApoH [141]. Gause et al. showed that a BSA (bovine serum albumin) corona decreases uptake by dendritic cells [142]. The denaturation of protein triggers innate immune activation through scavenger receptor A, a receptor for damaged or modified proteins [31].

These strategies must be read alongside significant challenges in reproducibility. In controlled multi‐center studies, in which the same materials were analyzed in different laboratories, the proteins consistently identified across facilities were a small minority of those reported, which indicates that some of the reported corona variation may reflect experimental noise rather than exploitable biological signal [75]. Batch‐to‐batch variability between nanoparticles made by nominally identical protocols compounds this uncertainty [143]. Corona‐aware design is therefore promising, but it faces a ceiling: the irreducible biological variability that must be quantified before optimization is possible.

#### 5.3.2 Active Targeting and Biomimetic Strategies

Figure 10C shows hard corona (HC), soft corona (SC), and complete corona interface (CI) shaping nanoparticle fate in vivo along different paths [44]. Time‐course analysis of copper content in blood, liver, and spleen from 3 to 360 min post‐injection separates the pharmacokinetic profiles by corona composition. Chirality registers here as well, with D‐NPs and L‐NPs forming stereospecific coronas and diverging in biodistribution. Dysopsonin engineering exploits stealth proteins directly, and pre‐coating with clusterin and ApoA1 maintained long circulation across both mouse and human serum [65]. Molecular imprinting creates albumin‐selective binding sites [144]. Patient‐specific precision nanomedicine goes furthest, using personalized coronas: nanoparticles pre‐coated in an individual patient's plasma proteins showed tumor‐specific accumulation that tracked treatment response [58].

Active targeting is where ligand presentation defeats a great deal of rational design. Herda et al. found only ≈3.5% of the transferrin grafted onto silica nanoparticle surfaces adopting an orientation favorable for receptor recognition, with the remainder presented in configurations unable to engage the receptor [66]. That orientation problem helps explain why so many actively‐targeted nanoparticles fail to beat passive EPR‐based accumulation despite sophisticated surface engineering. “Trojan horse” approaches invert the problem by co‐opting corona formation instead of fighting it. Engineer a surface to recruit brain‐targeting apolipoproteins and you get transcytosis across the barrier through their cognate receptors, with ApoE acting through LRP1, ApoJ through LRP2 and ApoA1 through SR‐B1, reaching 14.5‐fold higher brain distribution of the delivered drug than the unmodified liposome [133, 145]. Figure 10D makes the case visually, with CD31/DiI immunofluorescence imaging of brain and glioma tissue showing a 45‐fold increase in accumulation within the glioma region using SP‐sLip formulations built on apolipoprotein corona engineering [133]. Zhang et al. reported median survival extended from 16.5 to 45 days using ApoE‐enriched coronas [146]. Caracciolo et al. showed apolipoprotein coronas switching uptake pathways from caveolin‐ to clathrin‐mediated endocytosis, which opens nuclear delivery of therapeutic cargo [147]. Taken together these strategies represent a conceptual inversion, harnessing the biological identity transformation instead of resisting it.

Cell membrane coating transfers biological identity wholesale. Erythrocyte membranes, which present CD47 and other self‐markers, substantially extend nanoparticle circulation relative to synthetic stealth coatings, and cancer cell membranes have been used to confer homotypic tumor targeting. Scalable manufacturing and quality control for membrane‐coated formulations remain open problems inside DMTBL workflows.

### 5.4 Pillar IV: Living Systems Integration

Living systems integration anchors the whole DMTBL cycle in human‐relevant biology. This is the pillar that produces the evidence models must eventually match, using organ‐chips, organoids, and small organisms to approximate patient responses before anyone goes into humans.

#### 5.4.1 Automated High‐Content Screening and Organ‐on‐Chip Systems

Automated cellular platforms compress screening timelines. CellXpress.ai integrates robotic liquid handling, automated imaging, and environmental control, processing over 100 plates in parallel in its largest incubator configurations and increasing organoid production by up to 25‐fold [148]. High‐content screening quantifies multiparametric phenotypes at single‐cell resolution across 10,000–100,000 cells per well. Organ‐on‐chip platforms reproduce what traditional cultures leave out: tissue–tissue interfaces, mechanical microenvironments, and physiological flow. The limitation they answer is basic, since static 2D monolayers cannot replicate the dynamic three‐dimensional architecture and biomechanical forces governing drug transport and toxicity in living tissue. Figure 7B summarizes four platforms with nanomedicine‐relevant capabilities. The Lung‐Chip reconstitutes the alveolar‐capillary interface with cyclic mechanical stretch (10% strain, 0.2 Hz) mimicking breathing, and it revealed that mechanical strain increased silica nanoparticle translocation through mechanosensitive ion channel activation, a phenomenon entirely invisible in static Transwell cultures [56]. That result matters for inhaled nanomedicine development, where breathing mechanics fundamentally alter particle transport.

Hepatotoxicity prediction is where the Liver‐Chip proved itself, reaching 87% sensitivity and 100% specificity and correctly flagging drugs that injured human livers while appearing safe in animal tests [68]. It combines hepatocytes and sinusoidal endothelial cells in a configuration mimicking hepatic sinusoid architecture, with continuous perfusion maintaining metabolic function for weeks, which for nanomedicine means assessing nanoparticle‐induced hepatotoxicity under conditions that recapitulate first‐pass metabolism and MPS‐mediated clearance. A microengineered blood–brain barrier platform combining human brain microvascular endothelial cells, pericytes, and astrocytes has been used to resolve nanoparticle transport mechanisms, demonstrating SR‐B1‐mediated transcytosis of HDL‐mimetic nanoparticles across the barrier [149]. A fully 3D‐printed tumor‐on‐a‐chip supporting patient‐derived multicellular spheroids has been used for multi‐drug screening with correlation to clinical outcomes [150]. Cancer‐on‐chip models incorporating T cells, macrophages, and NK cells let immunomodulatory nanoparticles be tested in tumor microenvironments carrying human immune components [151].

FDA Modernization Act 2.0 (2022) removed a statutory requirement for animal testing in certain contexts and widened the pathway for non‐animal methods, an important step toward broader evaluation of microphysiological systems [152]. Policy has continued to move in this direction. The FDA's April 2025 roadmap for reducing animal testing in preclinical safety studies sets a three‐to‐five‐year horizon for integrated safety assessment built on new approach methodologies, beginning with monoclonal antibodies and expanding to other biologics and new chemical entities [153]. Legislation extending the 2022 reforms is advancing but is not yet enacted: the Senate passed S. 355 in December 2025 and the House passed the companion bill H.R. 2821 on July 20, 2026, with final congressional action still pending as of August 2026 [154]. Ingber reviewed organ‐chips for disease modeling and personalized medicine, documenting more than 15 organ systems now commercially available [155].

#### 5.4.2 Patient‐Derived Organoids and Model Organism Screening

Tumor organoids are small three‐dimensional cultures grown from a patient's own tumor tissue and tumor architecture, genetic heterogeneity, and drug response profiles are retained in them even though they are handled in 384‐well formats (Figure 7C). Ji et al. established 65 liver cancer organoid lines and identified mTOR pathway alterations that predict lenvatinib sensitivity [78]. Patient‐derived organoids have been reported to model treatment response in metastatic gastrointestinal cancers [69]. Organoids can also be derived from small biopsy specimens, shortening the interval between sampling and drug testing. Testing nanoparticles in these systems can reveal size‐dependent penetration behavior that is not apparent in monolayer culture. Organoid generation, treatment, and multi‐parametric imaging are now integrated on automated platforms. Patient‐derived xenografts, in which a fragment of the patient tumor is grown in a mouse, also preserve tumor architecture, but 3–6 months are required for establishment, and the immune component is absent unless humanized mice are used. Zebrafish and *C. elegans* serve as Tier 1 rapid whole‐organism screening platforms within a tiered validation hierarchy (Figure 7A). This review emphasizes species‐specific differences (Section 1.1), and yet it includes organisms more distant from humans than mice are, which needs some explanation. These organisms are used for different questions than organ‐chips and organoids are. They are *not* human‐relevant for protein corona composition or for immune specificity, and those endpoints require organ‐chips at Tier 2 and patient organoids at Tier 3. They are used instead for rapid whole‐organism toxicity screening, for biodistribution imaging, and for the clearance kinetics of the mononuclear phagocyte system, which is the network of resident macrophages that strips foreign particles out of the circulation, and none of these endpoints can be captured by isolated organ‐chip systems [79]. Approximately 70% of human genes have at least one obvious zebrafish orthologue [156], and zebrafish possess a conserved innate immune system with macrophage phenotypes comparable to those of mammals. Their high fecundity and optical transparency allow large numbers of embryos to be screened in parallel with biodistribution visualized in real time [79]. Transgenic reporter lines, in which a fluorescent protein is expressed in one cell type only, allow clearance of nanoparticles by the mononuclear phagocyte system to be watched in vivo, with Tg(fli1:EGFP) labeling the vasculature and Tg(mpeg1:mCherry) labeling the macrophages. *C. elegans*, a 1 mm soil nematode, offers genetic tractability together with 60%–80% homology to human disease genes; QuantWorm systems have been used to screen 20 nanomaterials across four concentrations for body length, locomotion, and lifespan endpoints [80], microfluidic “worm chips” allow nanoparticle uptake and stress reporter activation to be imaged in real time [157], and CRISPR knockout libraries are used for mechanism‐of‐action work. Within the DMTBL framework, model organisms therefore occupy a specific niche, which is the rapid elimination of overtly toxic candidates, a matter of days, before scarce organ‐chip and organoid resources are committed to the most promising formulations, a matter of weeks.

#### 5.4.3 Limitations of Human‐Relevant Platforms

Despite their advantages, current human‐relevant validation platforms have important limitations that constrain how well they fit into DMTBL workflows. First, adaptive immune components (T cells, B cells, and mature dendritic cells) are absent from most organ‐chip systems, and this is a critical gap for the evaluation of immunomodulatory nanoparticles in immuno‐oncology applications. Second, systemic multi‐organ pharmacokinetic interactions cannot be captured by single‐organ chips, since clearance by the mononuclear phagocyte system in the liver reduces the dose that reaches downstream target organs, and this effect is invisible in isolated organ‐chip configurations. Third, the fundamental training data for predictive models, meaning quantitative measurements of nanoparticle accumulation in human tumors, barely exists, because intratumoral drug concentration is not measured in most clinical nanomedicine trials; bridging strategies include imaging‐based surrogates (PET tracers with radiolabeled nanoparticles), microdialysis in accessible tumors, and pharmacokinetic analysis of surgical specimens. Fourth, inter‐platform reproducibility remains a significant challenge, and inter‐laboratory variation in organoid viability testing in China has been reported at 40% [70], with variations of up to 30% in first‐pass metabolism rate observed across multi‐organ chip configurations [71]. That last point is the most damaging of the four. These levels of variability can exceed the formulation‐dependent signal that DMTBL is meant to optimize, so standardized protocols and reference materials are needed before organ‐chip data can reliably close the optimization loop.

### 5.5 Implementation: Investment, Standards, and Regulatory Pathways

Implementation depends as much on data standards and on regulatory acceptance as it does on hardware, though the hardware is where the numbers are clearest. Basic DMTBL nodes ($150–$250K) integrate the essential automation, which comprises OpenTrons liquid handlers ($5–$20K), microfluidic synthesis platforms ($30–$50K), environmental chambers ($15–$25K), and automated imaging ($80–$120K) [17, 54]. Open‐source and 3D‐printed alternatives substantially reduce the cost of entry relative to commercial systems [158]. Full‐stack platforms ($500K–$1.2M) add organ‐chips ($50–$150K) and zebrafish automation ($200–$400K), and distributed networks ($2–$5M per site) establish specialized nodes connected through federated learning architectures, in which a shared model is trained across institutions without the underlying data leaving any single site [159]. On the data side, workflow orchestration is handled by Snakemake or Nextflow, which are pipeline managers that record and re‐run every computational step, with containerization to keep results consistent [17]. The FAIR principles, meaning findable, accessible, interoperable, and reusable, mandate persistent identifiers, controlled vocabularies, and open formats [73], while the ISA framework, standing for Investigation, Study, and Assay, provides structured metadata templates. Biology demands version control of its own, and here the requirements are more specific than for software: cell lines require periodic STR profiling, that is, short tandem repeat fingerprinting to confirm identity; organoid versioning tracks differentiation protocols and quality metrics; and organ‐chip reproducibility requires CAD files and written standard operating procedures. Physiologically based pharmacokinetic (PBPK) modeling is already recognized by both the FDA and the EMA for nanomedicine submissions [152, 160]. Validation of AI and ML models requires explainable techniques such as SHAP values, LIME, and attention mechanisms, all of which identify which input features drove a given prediction [161]. Regulatory pathways also require demonstrated manufacturing consistency, for which a coefficient of variation below 5% is a commonly applied in‐house target rather than a universal specification; analytical method validation per ICH Q2(R2); preclinical safety packages that integrate organ‐chip data; and clinical validation through adaptive trial designs that use Bayesian statistics and real‐world evidence.

## 6 Roadmap: Autonomous Nanomedicine 2030

### 6.1 The Convergence Vision

Integration of the four pillars within DMTBL frameworks could, if the integration challenges documented in Sections 3.2– 3.3 are resolved, substantially advance nanomedicine from empirical discovery toward more predictive optimization [52]. The value would come from combining automated speed with human relevance, since organ‐chips supplement and in some cases replace animal models, and with computational intelligence, since candidates can be ranked by AI before they are synthesized. Closed‐loop integration nonetheless remains at Technology Readiness Level (TRL) 2–3, meaning proof of concept rather than a demonstrated working system, despite the maturity of the individual components. The timeline estimates below are contingent on progress across several independent technical and regulatory fronts, and they should be read as aspirational targets rather than as projections.

### 6.2 Timeline and Milestones

#### 6.2.1 Short‐Term (2025–2028): Foundation Building

The near‐term work is mostly consolidation. Nano‐QSAR training sets, meaning quantitative structure–activity relationship models fitted to nanomaterials, could plausibly expand by an order of magnitude through federated or swarm learning consortia of the kind demonstrated for decentralized, confidentiality‐preserving clinical machine learning [159], and validated PBPK models may become accepted by regulators as FDA and EMA guidance is implemented [152, 160]. Commercial organ‐chips could reach ISO 13485/10993 standardization with an inter‐laboratory coefficient of variation below 15%, automated organoid platforms may gain 10–100× throughput through robotic integration [148], ML‐guided corona engineering could produce species‐invariant formulations [65], and open‐source self‐driving‐laboratory (SDL) software may bring entry costs below $50K [158]. Realistically, prospective validation should be expected to perform well below the retrospective figures summarized in Table 2, with organ‐chip‐to‐clinical correlation studies enrolling 500+ patients and partial DMTBL demonstrations in which biological feedback from 2D culture updates the generative models.

#### 6.2.2 Mid‐Term (2028–2032): Clinical Integration

Achievable milestones include the transition of patient organoid screening into clinical decision support, with a turnaround of 2–3 weeks. Multi‐organ‐chip systems would be used to model systemic pharmacology, with liver‐tumor‐kidney configurations, gut‐liver chips for oral bioavailability, and heart‐liver chips for detecting synergies between cardiotoxicity and hepatotoxicity, while semi‐autonomous platforms would execute 100+ formulation‐biology cycles monthly. Regulatory acceptance of organ‐chip efficacy data as a supplement to animal studies is possible in this window. Realistic expectations include Phase 2 success rates improving from 8%–12% to 15%–20% for organoid‐selected populations, regulatory guidance that specifies organ‐chip acceptance criteria, and a first demonstration that DMTBL‐optimized formulations outperform conventional comparators.

#### 6.2.3 Long‐Term (2032+): Precision Autonomy

Beyond 2032 the projections become genuinely speculative. More highly autonomous nanomedicine discovery could compress workflows far enough to make precision medicine approaches practical, and an envisioned biopsy‐to‐treatment pipeline might run as follows: tumor biopsy (day 0) → organoid establishment and expansion (days 1–14) → AI‐designed formulation library targeting patient‐specific corona composition, tumor markers, and genomic features (days 15–16) → robotic microfluidic synthesis and characterization (day 17) → organoid efficacy and toxicity screening with multi‐parametric readouts (days 18–25) → lead optimization through additional DMTBL iterations (days 26–30) → GMP manufacturing and clinical administration (days 31–60). Formulations could then be matched to the proteome, the genomics, and the disease state of the individual patient, though substantial technical and regulatory hurdles remain. Clinical success rates might approach those of small molecules, which run at 10%–15% from Phase 1 to approval, against the current 3%–4%, if human‐relevant preclinical models and computational prediction reduce clinical attrition as anticipated. Human microphysiological systems may substantially supplement, and in some cases replace, animal testing for efficacy and toxicity assessment, with animals potentially reserved for complex systemic endpoints such as behavioral toxicity, reproduction, and carcinogenicity, all of which require years‐long observation. Distributed global networks of autonomous DMTBL nodes could speed pandemic response, though the practical timeline for such developments remains uncertain. Critical dependencies include: large‐scale prospective validation showing that organ‐chip and organoid predictions correlate with clinical outcomes across diverse drug classes; regulatory harmonization across the FDA, the EMA, and the PMDA of Japan, accepting computational and microphysiological evidence with standardized qualification criteria; cost reduction sufficient to make routine clinical use possible; and demonstrated closed‐loop integration in which biological feedback from human tissue models actually closes the optimization loop.

### 6.3 Limitations and Open Questions

Several limitations and open questions remain alongside the opportunities that have been outlined in this review. First, organoid systems are not yet standardized to the degree that would be required for routine use in regulatory decision‐making. Consensus protocols, reference materials, and inter‐laboratory proficiency testing programs, in which identical samples are analyzed at several sites and the results compared, are needed if the current inter‐site variability is to be reduced, since that variability remains above the commonly accepted threshold of 15% coefficient of variation (CV), which is the standard deviation of a measurement expressed as a fraction of its mean. Until such standardization is in place, results will be difficult to compare across centers, and they will be difficult to aggregate at the scale that model training requires. This is a precondition for most of what follows, since a model can be no more reliable than the assay variability underlying the data it is fitted to. Second, the translational evidence base for organ‐on‐chip platforms, in which human cells are cultured under perfusion in microfabricated channels, is still narrow. Most prospective clinical correlation studies have been focused on hepatotoxicity, meaning drug‐induced injury to the liver, and impressive predictive performance has been reported there, while the analogous data for efficacy endpoints and for other organ systems remain sparse. Extension of these studies to broader drug classes, to more diverse patient populations, and to longitudinal clinical outcomes will require substantial investment and coordinated consortia. Third, the artificial intelligence models used within DMTBL pipelines must satisfy emerging expectations for interpretability. Regulatory acceptance will depend on whether model predictions can be linked back to mechanistic principles, rather than on opaque statistical correlations alone [152]. Methods that provide mechanistic feature attributions, that quantify the uncertainty attached to each prediction, and that perform robustly across population subgroups will therefore be essential if confidence is to be built among regulators and clinicians. Fourth, the failure modes of closed‐loop optimization are not yet fully characterized. Autonomous systems can converge on local optima, meaning solutions that cannot be improved by small changes but are not the best available overall, or they can amplify systematic biases that are already present in the training data, and this happens particularly when the performance metrics being optimized are narrowly defined. The gap between training and validation performance seen across the prediction tasks compared here (Table 2) likely reflects memorization of dataset‐specific biases, and biases of that kind would propagate through iterative optimization cycles rather than being corrected by them. Applicability domain violations, in which a model is asked to predict for regions of chemical space that lie outside its training distribution, are not routinely assessed in nanomedicine machine learning, and no standard test for them is in common use, so there is a real risk that autonomous systems will confidently propose formulations for which their own predictions are unreliable. A key open question is how to detect and mitigate the cases in which iterative optimization consistently favors formulations that are systematically biased with respect to particular patient subgroups, anatomical sites, or clinical endpoints. Explicit safeguards will be required, and these include held‐out validation sets that are never seen during training and that are refreshed with each optimization iteration, bias audits carried out against subgroup definitions fixed in advance, and human‐in‐the‐loop oversight at defined decision gates. Fifth, protein corona variability may be a more fundamental obstacle than is sometimes acknowledged. Patient‐specific factors, among them biological sex, genetic ancestry, disease state, environmental exposures, and age, all produce corona compositions that differ both qualitatively and quantitatively [143]. In controlled studies, the proteins consistently identified across independent facilities were a small minority of those reported, which suggests that some of the reported corona variation may reflect experimental noise rather than a true biological signal [75]. Batch‐to‐batch nanoparticle variability, arising even from nominally identical synthesis protocols, adds a further layer of uncertainty that DMTBL models must navigate. Corona‐aware design is therefore promising, but it faces a ceiling that is imposed by irreducible biological variability, and standardization alone may not be sufficient to overcome it, since some of the variability is a property of the patients rather than of the protocol. Sixth, the health economics of DMTBL implementation remain incompletely quantified. Investment tiers and potential efficiency gains have been outlined in the preceding sections, but formal cost‐effectiveness analyses that compare DMTBL‐enabled workflows against traditional nanomedicine development pipelines are still needed. Such analyses should incorporate not only the direct research and development costs but also the downstream effects on trial success rates, on time to approval, and on patient outcomes. Finally, questions of access and equity are central to all of this. Automation and microphysiological infrastructure are capital‐intensive, and there is a risk that capabilities will be concentrated in a small number of well‐resourced institutions as a result, since the entry cost alone excludes most groups that would otherwise contribute. Without deliberate international investment, shared platforms, and capacity‐building initiatives, there is a real danger that autonomous nanomedicine will widen the existing gaps between high‐income settings and low‐ and middle‐income settings rather than closing them.

## 7 Conclusion: A Research Agenda With Realistic Timelines

The nanomedicine translation crisis is exemplified by a median tumor delivery of 0.67% of the injected dose in preclinical solid‐tumor models that has not improved significantly over 16 years of research [1, 3], and by persistently low clinical success rates. Inadequate biological validation is a major and under‐addressed contributor to that crisis, distinct from the physicochemical design considerations that have received far more attention. Nanoparticles that have been optimized for size, surface charge, and encapsulation efficiency frequently fail in the clinic, because their behavior is transformed by the protein corona, shaped by species‐specific immune responses, constrained by heterogeneous enhanced permeability and retention (EPR), which is the leaky‐vasculature effect that passive tumor targeting depends on, and governed by emergent multi‐scale phenomena that traditional preclinical models do not capture. There is also a pronounced asymmetry between fully automated synthesis and largely manual, low‐throughput biological validation, and it is that asymmetry, rather than any shortage of candidate formulations, which has become the rate‐limiting step in translation. Autonomous nanomedicine aims to resolve this bottleneck by embedding human‐relevant biological testing throughout discovery, instead of relying on late‐stage animal‐centric validation. The central promise is improved clinical predictivity: organ‐on‐chip platforms achieving a sensitivity of 87%, meaning the fraction of genuinely toxic compounds that are correctly flagged, and a specificity of 100%, meaning the fraction of safe compounds that are correctly cleared, for hepatotoxicity [68] (Figure 7B), patient‐derived organoids whose responses have been reported to track clinical outcomes (Figure 7C), and physiologically based pharmacokinetic (PBPK) models that account for much of the variance in measured delivery on their training data [36]. Taken together, these advances represent a shift away from preclinical pipelines whose concordance with human outcomes is incomplete [77], and toward human‐centric models that are explicitly calibrated against patient data. Three aspects of the contribution made here are worth emphasizing. The DMTBL framework formally separates “Biology” from “Test” as a distinct optimization node, and this creates a closed‐loop feedback mechanism running from human‐relevant biological performance back to AI‐guided design, which the traditional DMTA cycle leaves implicit, since its analysis step is in most implementations applied to chemical and physical measurements alone. The technology readiness level (TRL) assessment (Table 1) then provides explicit, author‐assigned criteria adapted for biomedical contexts, and it reveals that Pillar I (Predictive Intelligence) operates at TRL 3–5 rather than at the higher levels that the reported performance metrics might suggest, a distinction that is driven by the rarity of prospective validation, meaning validation carried out on predictions registered before the outcome was known rather than on data already in hand. A third contribution is the specification of measurable convergence targets for closing the integration gap, and those targets are set out in the section that follows. Together they convert the general observation that integration lags behind component maturity into a testable research agenda.

### 7.1 Priority Research Investments

Several research and infrastructure investments follow from these considerations. First, large prospective validation studies enrolling more than 500 patients are needed, so that the degree to which organ‐chip and organoid responses predict clinical outcomes can be quantified rigorously across diverse drug classes, disease indications, and patient subgroups. Second, federated learning consortia, in which a shared model is trained across institutions without proprietary data leaving any single site, would allow nano‐QSAR datasets, meaning quantitative structure‐activity relationship models fitted to nanomaterials, and related datasets to be aggregated from the current 3000 formulations to scales of 50,000 or more, and this would improve model generalizability and reduce the institutional silos that currently keep comparable datasets apart. Third, coordinated efforts toward ISO‐style standardization of organ‐chip workflows, including agreed definitions of performance metrics and inter‐laboratory proficiency testing schemes, should target inter‐site variability below 10% coefficient of variation, against the 40% inter‐laboratory variation reported for organoid viability testing. Fourth, structured regulatory harmonization efforts involving agencies such as the FDA, the EMA, and the PMDA of Japan are needed, so that acceptance criteria for computational models and for microphysiological systems, which are engineered tissue constructs that reproduce organ‐level function, can be defined, evidentiary standards clarified, and uncertainty for developers reduced. Finally, open‐source self‐driving‐laboratory (SDL) platforms, meaning automated systems that plan and execute their own experiments, together with reference implementations that bring the total cost of entry below approximately $50,000, would democratize access, so that smaller institutions and international collaborators are able to participate in autonomous nanomedicine research rather than remaining dependent on a few centralized hubs. The realistic horizon for transformative clinical impact, on the evidence assembled here, is on the order of 2030–2035. By that point, the success of autonomous nanomedicine should be evaluated not only by economic metrics but also by the number of lives improved through accelerated pandemic responses, by the viability of formulations targeting historically neglected diseases, and by the routine deployment of precision nanomedicines that are matched to individual patients through organoid and organ‐chip predictions. If it is pursued with intellectual honesty about the current limitations, and with a systematic program to address them, autonomous nanomedicine can convert the 0.7% delivery problem from a seemingly intractable limitation into an engineering challenge that can be progressively quantified, optimized, and ultimately overcome.

## Author Contributions

**Yan Jun Lin**: conceptualization, methodology, software, formal analysis, investigation, data curation, writing – original draft, visualization. **Izabella Komperda**: conceptualization, methodology, writing – review and editing, supervision.

## Funding

The authors have nothing to report.

## Conflicts of Interest

The authors declare no conflicts of interest.

## Data Availability

Data sharing not applicable to this article as no datasets were generated or analyzed during the current study.
